# Supplementary material for: Global patterns of plant functional traits and their relationships to climate
Source: Commun Biol. 2024 Sep 13;7:1136. doi: 10.1038/s42003-024-06777-3 (PMC11399309; doi:10.1038/s42003-024-06777-3)
Supplement: Supplementary file 2 — Supplementary Information [file 42003_2024_6777_MOESM2_ESM.pdf]

Global patterns of plant functional traits and their relationships to climate

Jiaze Li[1], Iain Colin Prentice[1,2]

[1]Georgina Mace Centre for the Living Planet, Department of Life Sciences, Imperial College London, Silwood Park Campus, Buckhurst Road, Ascot, SL5 7PY, UK

[2]Department of Earth System Science, Ministry of Education Key Laboratory for Earth System Modeling, Institute for Global Change Studies, Tsinghua University, Beijing 100084, China

Corresponding author: Jiaze Li (email: jiaze.li19@imperial.ac.uk)

Supplementary Information

SI 1 Analysis for 16 plant functional traits

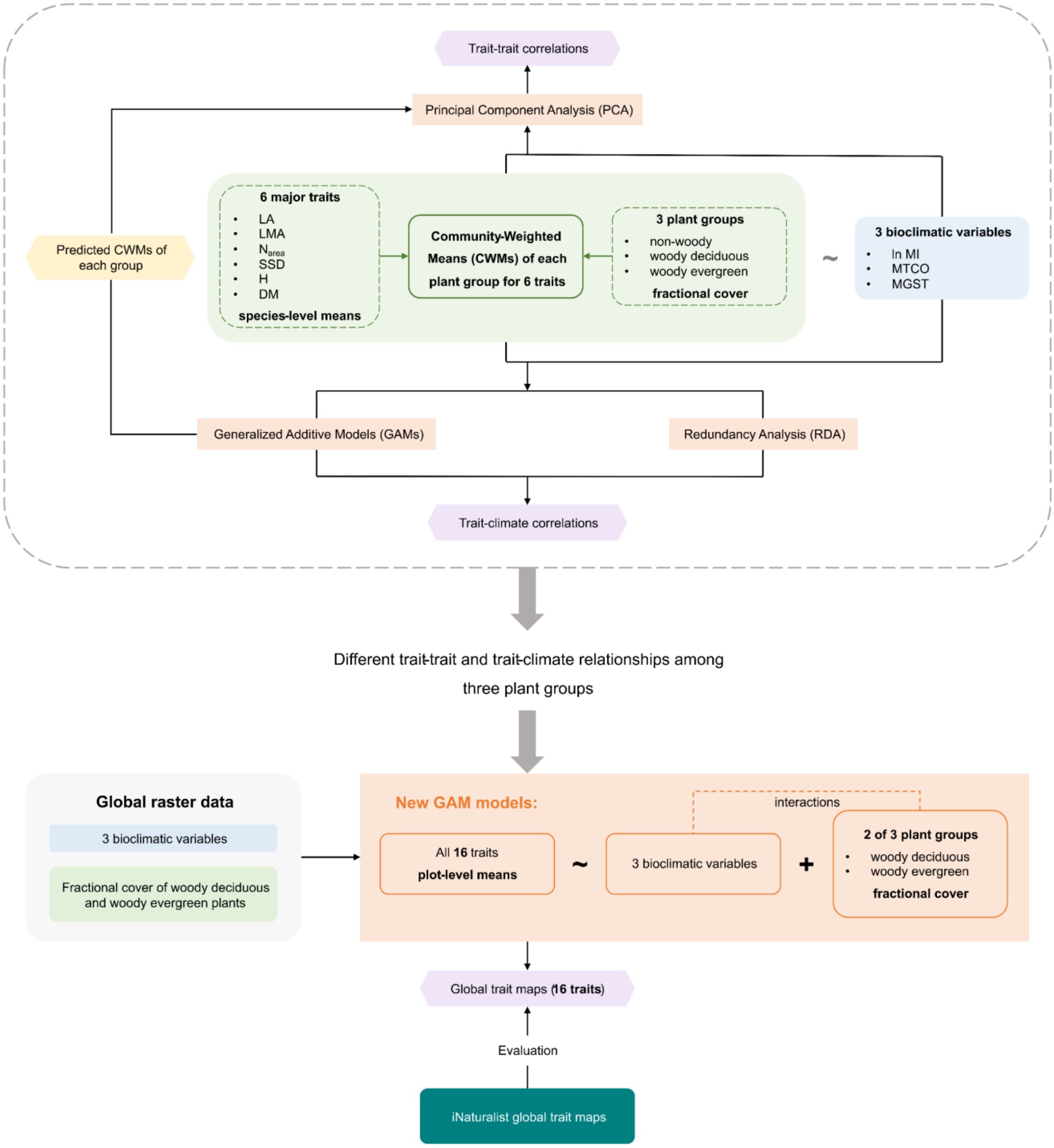

Fig. S1 | Overview of the study. All elements of the figure were created by the authors.

Table S1 | Sample size of all data used in this study. [a] Plant functional traits used in this study. [b] Abbreviation and [c] unit of each of the 16 traits. [d] Number of plots having plot-level mean trait values. [e] Number of species having species-level mean trait values. [f] Number of pixels of iNaturalist trait maps at 2° spatial resolution. [g–h] Number of pixels of global trait maps upscaled from generalised additive models (GAMs) at [g] 0.1° spatial resolution and [h] 2° spatial resolution. [i] Number of pixels having both iNaturalist trait values and GAM trait values when making comparisons at 2° spatial resolution. The first six traits are major traits having both species-level[1] and plot-level trait[2] means. The remaining traits only have plot-level trait means (community-weighted means, CWMs)[2].

Trait[a]	Abbreviation[b]	Unit[c]	N_{plot}[d]	$N_{species}$[e]	$N_{iNaturalist}$[f]	N_{GAM01}[g]	N_{GAM2}[h]	$N_{Comparison}$[i]
Leaf area	LA	mm^2			3,381			1,851
Leaf mass per unit area	LMA	kg/m^2			3,573			1,916
Leaf nitrogen content per unit area	N_{area}	g/m^2		35,773	3,433			1,871
Stem specific density	SSD	g/cm^3			3,198			1,802
Plant height	H	m			3,732			1,980
Diaspore mass	DM	mg			3,749			1,987
Leaf fresh mass	LFM	g			2,476			1,417
Leaf phosphorus content per unit area	P_{area}	g/m^2			3,421			1,856
Leaf carbon content	C_{mass}	mg/g	77,074		3,326	830,086	2,397	1,818
Leaf dry matter content	LDMC	g/g			3,286			1,800
Stem conduit (vessel and tracheid) element length	WVL	µm		0	2,314			1,320
Stem conduit density	SCD	mm^{-2}			2,869			1,622
Seed number per reproductive unit	SN	NA			2,707			1,502
Seed length	SL	mm			2,745			1,528
Dispersal unit length	DUL	mm			2,818			1,534
Leaf nitrogen isotope ratio	$\delta^{15}N$	per million			2,774			1,602

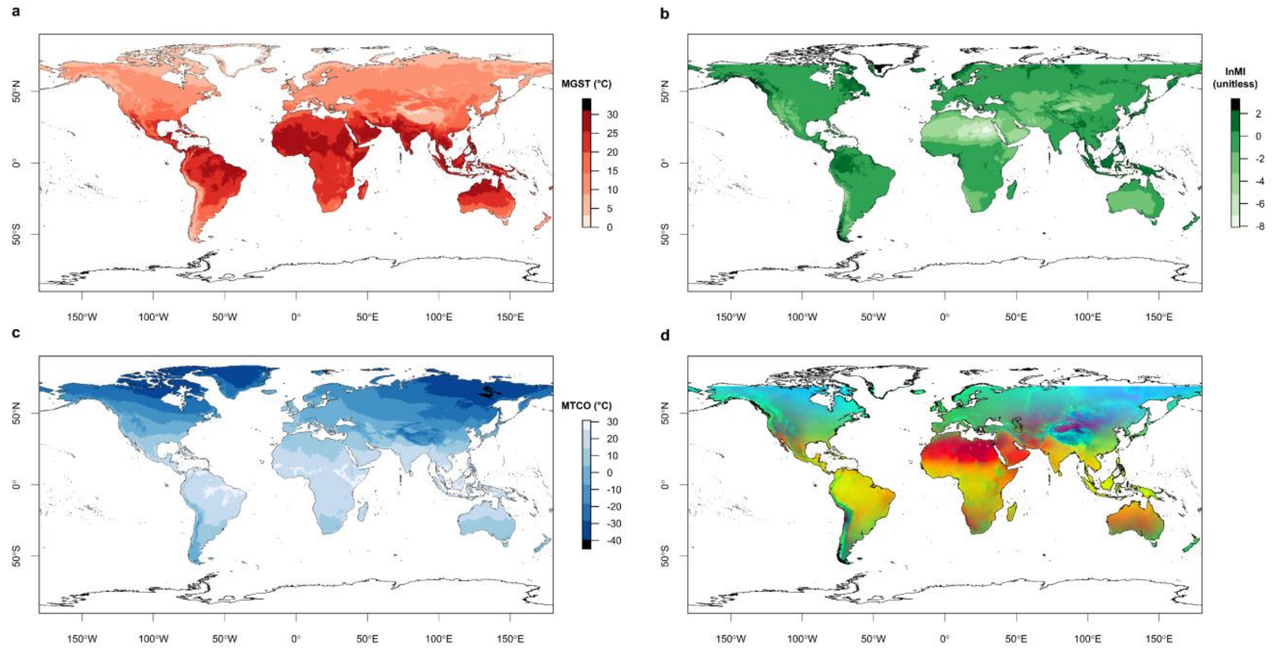

Fig. S2 | Global climates and global climate classes. a – c, Gridded global distribution of three bioclimatic variables at 0.1° spatial resolutions: **a**, mean growing-season temperature (MGST, ℃); **b**, natural-log transformed moisture index (ln MI, unitless); **c**, mean temperature of the coldest month (MTCO, ℃). **d**, map of global climate classes. The global climate classes were defined by three bioclimatic variables as follows: the grid values of each bioclimatic raster were rescaled into 0 – 255 and were then combined as RGB colour codes with red representing MGST, green representing ln MI and blue representing MTCO by the 'plotRGB()' function in R *raster* package[3].

Table S2 | Recorded plants and their relative proportion in the plot-level trait dataset. [a] Categories of recorded plants from the sPlotOpen database. [b] Number of plots recorded in each category in the plot-level trait dataset . [c] Proportion of each category to all records in the plot-level trait dataset.

Recorded plants[a]	N_{plot}[b]	Proportion[c]
All trees & dominant understory	110	0.14%
All vascular plants	12,743	16.53%
All woody plants	3,037	3.94%
Dominant trees	1	0.00%
Dominant woody plants >= 2.5 cm dbh	293	0.38%
Not specified	55,587	72.12%
Only dominant species	1,208	1.57%
Woody plants >= 1 cm dbh	14	0.02%
Woody plants >= 1 m height	24	0.03%
Woody plants >= 10 cm dbh	678	0.88%
Woody plants >= 2.5 cm dbh	2,837	3.68%
Woody plants >= 20 cm dbh	38	0.05%
Woody plants >= 5 cm dbh	504	0.65%

Table S3 | Summary of plant growth form dataset. [a] 16 plant growth forms defined based on the woodiness, plant growth form and leaf phenology of plants. [b] Brief description of each growth form. [c] Number of plant taxa within each growth form. [d] Proportion of plant taxa within each growth form to all plant taxa in the plant growth form dataset.

Plant growth form[a]	Description[b]	N_{taxa}[c]	Proportion[d]
non-woody	All non-woody taxa, e.g., herb, herbaceous vine…	21,483	50.34%
woody deciduous	All woody taxa with deciduous leaves; also including semi-deciduous or rarely/briefly evergreen taxa	3,659	8.57%
woody evergreen	All woody species with evergreen leaves; also including semi-evergreen or rarely/briefly deciduous taxa	9,385	21.99%
woody mix	All woody taxa with both deciduous and evergreen members	512	1.20%
woody NA	Woody plants with unavailable leaf phenology	5,083	11.91%
non-woody/woody deciduous	Taxa having both non-woody members and woody members with deciduous leaves	121	0.28%
non-woody/woody evergreen	Taxa having both non-woody members and woody members with evergreen leaves	151	0.35%
non-woody/woody mix	Taxa having both non-woody members and woody members with deciduous or evergreen leaves	58	0.14%
non-woody/woody NA	Taxa having both non-woody members and woody members with unavailable leaf phenology	198	0.46%
fern	All ferns	1,222	2.86%
bamboo	All bamboos	42	0.10%
palm	All palms	270	0.63%
cycad	All cycads	18	0.04%
succulent	All succulents	374	0.88%
cactus	All cacti	89	0.21%
NA	Lichen, liverworts, moss, clubmoss, fungi, algae…	11	0.03%

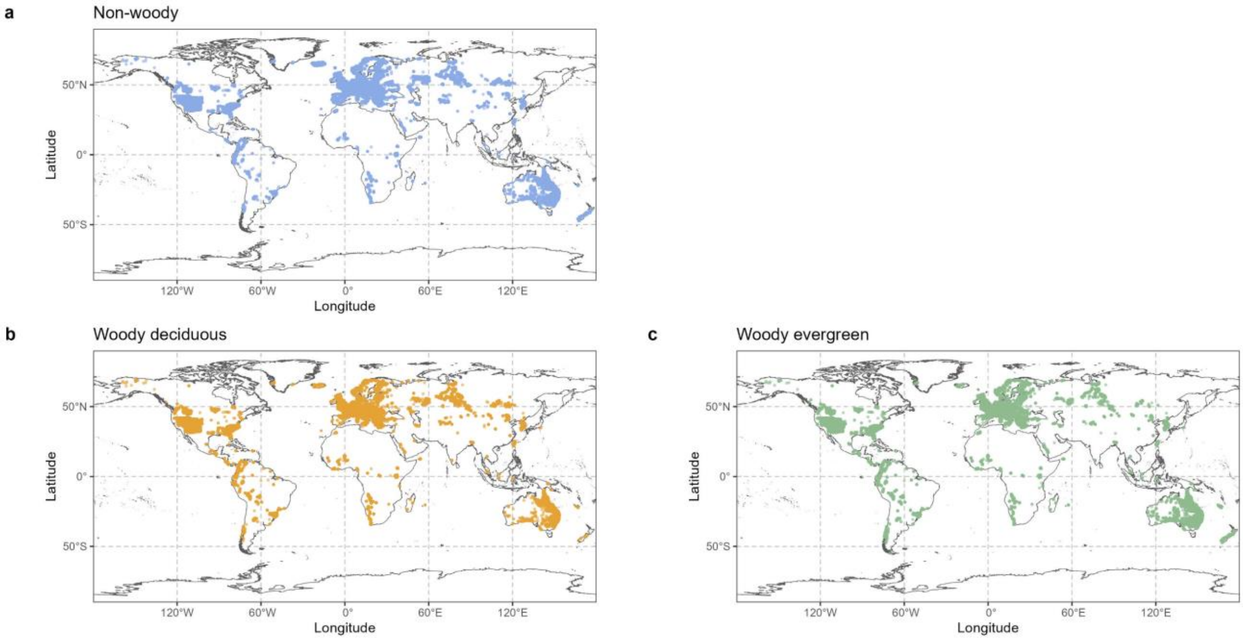

Fig. S3 | Global distributions of vegetation plots having community-weighted means (CWMs) of three plant groups for six major plant functional traits. a, global distribution of plots with trait CWMs of non-woody plants (number of plots: 66,066); **b**, global distribution of plots with trait CWMs of woody deciduous plants (number of plots: 46,659); **c**, global distribution of plots with trait CWMs of woody evergreen plants (number of plots: 52,802). All elements of the figure were created by the authors using R v4.2.2 (https://www.r-project.org/)[82].

Table S4 | Probability of each plant growth form being three plant groups. [a] 16 plant growth forms used in this study, see Table S3 for more information. [b] The probability that a growth form is assumed to be non-woody. [c] The probability that a growth form is assumed to be woody deciduous. [d] The probability that a growth form is assumed to be woody evergreen.

Plant growth form[a]	Probability of being non-woody[b]	Probability of being woody deciduous[c]	Probability of being woody evergreen[d]
non-woody	1	0	0
woody deciduous	0	1	0
woody evergreen	0	0	1
woody mix	0	0.5	0.5
woody NA	0	0.5	0.5
non-woody/woody deciduous	0.5	0.5	0
non-woody/woody evergreen	0.5	0	0.5
non-woody/woody mix	0.33	0.33	0.33
non-woody/woody NA	0.33	0.33	0.33
fern	0	0	0
bamboo	0	0	0
palm	0	0	0
cycad	0	0	0
succulent	0	0	0
cactus	0	0	0
NA	0	0	0

Table S5 | Fractional covers of three plant groups for all land cover classes and sub-classes. [a] 23 classes of global land cover in the ESA CCI-LC global land cover dataset. [b] 38 sub-classes of global land cover presented as grid values in the ESA CCI-LC maps. [c] Brief description of each land cover sub-class. [d] Fractional cover of non-woody plants in each land cover sub-class. [e] Fractional cover of woody deciduous plants in each land cover sub-class. [f] Fractional cover of woody evergreen plants in each land cover sub-class. The detailed calculation of global fractional covers of three plant groups are shown in Methods.

Class[a]	Sub-class[b]	Description[c]	Coverage of non-woody[d]	Coverage of woody deciduous[e]	Coverage of woody evergreen[f]
No data	0	No data	NA	NA	NA
Rainfed cropland	10	Cropland, rainfed	NA	NA	NA
	11	10 - Herbaceous cover	NA	NA	NA
	12	10 - Tree or shrub cover	NA	NA	NA
Irrigated or post-flooding cropland	20	Cropland, irrigated or post-flooding	NA	NA	NA
Mosaic cropland	30	Mosaic cropland (>50%) / natural vegetation (tree, shrub, herbaceous cover) (<50%)	0.7133	0.0163	0.2056
Mosaic natural vegetation	40	Mosaic natural vegetation (tree, shrub, herbaceous cover) (>50%) / cropland (<50%)	0.6092	0.0166	0.2521
Broadleaved evergreen tree cover	50	Tree cover, broadleaved, evergreen, closed to open (>15%)	0.3608	0.0215	0.5658
Broadleaved deciduous tree cover	60	Tree cover, broadleaved, deciduous, closed to open (>15%)	0.0655	0.7072	0.0983

	61	60 - Tree cover, broadleaved, deciduous, closed (>40%)	0.9730	0.0051	0.0180
	62	60 - Tree cover, broadleaved, deciduous, open (15-40%)	0.3535	0.0212	0.5315
Needleleaved evergreen tree cover	70	Tree cover, needleleaved, evergreen, closed to open (>15%)	0.3182	0.2431	0.2528
	71	70 - Tree cover, needleleaved, evergreen, closed (>40%)	NA	NA	NA
	72	70 - Tree cover, needleleaved, evergreen, open (15-40%)	NA	NA	NA
Needleleaved deciduous tree cover	80	Tree cover, needleleaved, deciduous, closed to open (>15%)	0.3091	0.2690	0.0783
	81	80 - Tree cover, needleleaved, deciduous, closed (>40%)	NA	NA	NA
	82	80 - Tree cover, needleleaved, deciduous, open (15-40%)	NA	NA	NA
Mixed-leaf-type tree cover	90	Tree cover, mixed leaf type (broadleaved and needleleaved)	0.0197	0.6726	0.2346
Mosaic woody cover	100	Mosaic tree and shrub (>50%) / herbaceous cover (<50%)	0.6212	0.0081	0.3014

Mosaic herbaceous cover	110	Mosaic herbaceous cover (>50%) / tree and shrub (<50%)	0.6519	0.0254	0.2462
Shrubland	120	Shrubland	0.5631	0.0241	0.2857
	121	120 - Evergreen shrubland	NA	NA	NA
	122	120 - Deciduous shrubland	0.5667	0.0119	0.3867
Grassland	130	Grassland	0.9356	0.0056	0.0242
Lichens and mosse	140	Lichens and mosses	NA	NA	NA
Sparse vegetation	150	Sparse vegetation (tree, shrub, herbaceous cover) (<15%)	0.6986	0.0085	0.2281
	151	150 - Sparse tree (<15%)	NA	NA	NA
	152	150 - Sparse shrub (<15%)	NA	NA	NA
	153	150 - Sparse herbaceous cover (<15%)	NA	NA	NA
Fresh/brackish-water-flooded tree cover	160	Tree cover, flooded, fresh or brackish water	0.1282	0.6121	0.1372
Saline-water-flooded tree cover	170	Tree cover, flooded, saline water	0.4492	0.0004	0.4974
Flooded shrub or herbaceous cover	180	Shrub or herbaceous cover, flooded, fresh/saline/brackish water	0.5843	0.0149	0.2801
Urban areas	190	Urban areas	NA	NA	NA
Bare areas	200	Bare areas	NA	NA	NA
	201	200 - Consolidated bare areas	NA	NA	NA

	202	200 - Unconsolidated bare areas	NA	NA	NA
Water bodies	210	Water bodies	NA	NA	NA
Permanent snow and ice	220	Permanent snow and ice	NA	NA	NA

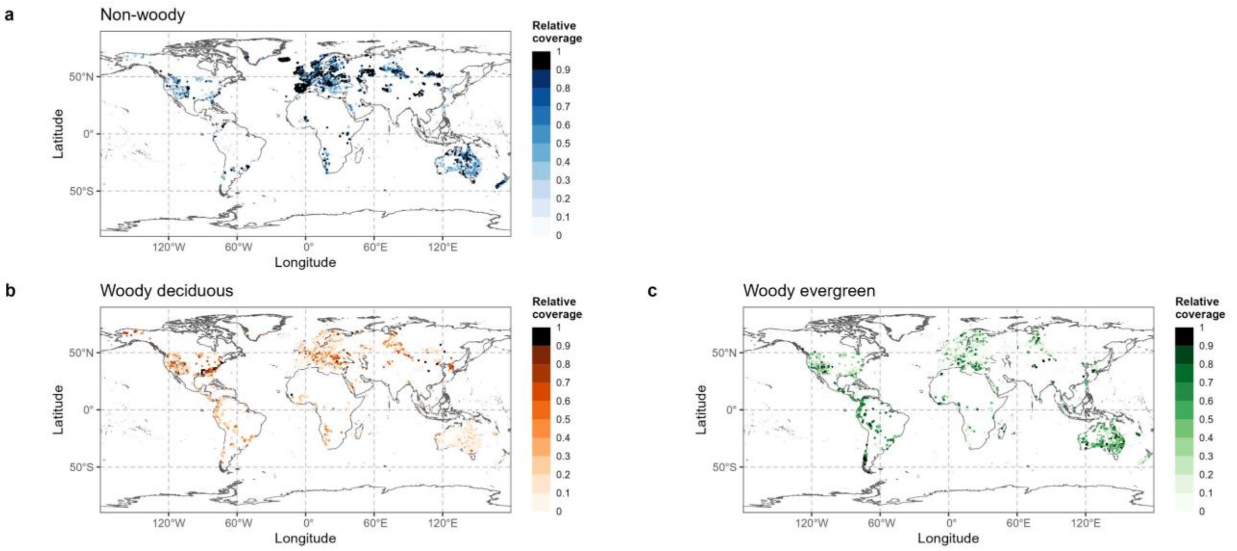

Fig. S4 | Relative coverages of three plant groups in all vegetation plots. Global distribution of all 77,074 natural vegetation plots used in this study and the relative coverage of non-woody plants (**a**), woody deciduous plants (**b**) and woody evergreen plants (**c**) within each plot. All elements of the figure were created by the authors using R v4.2.2 (https://www.r-project.org/)[82].

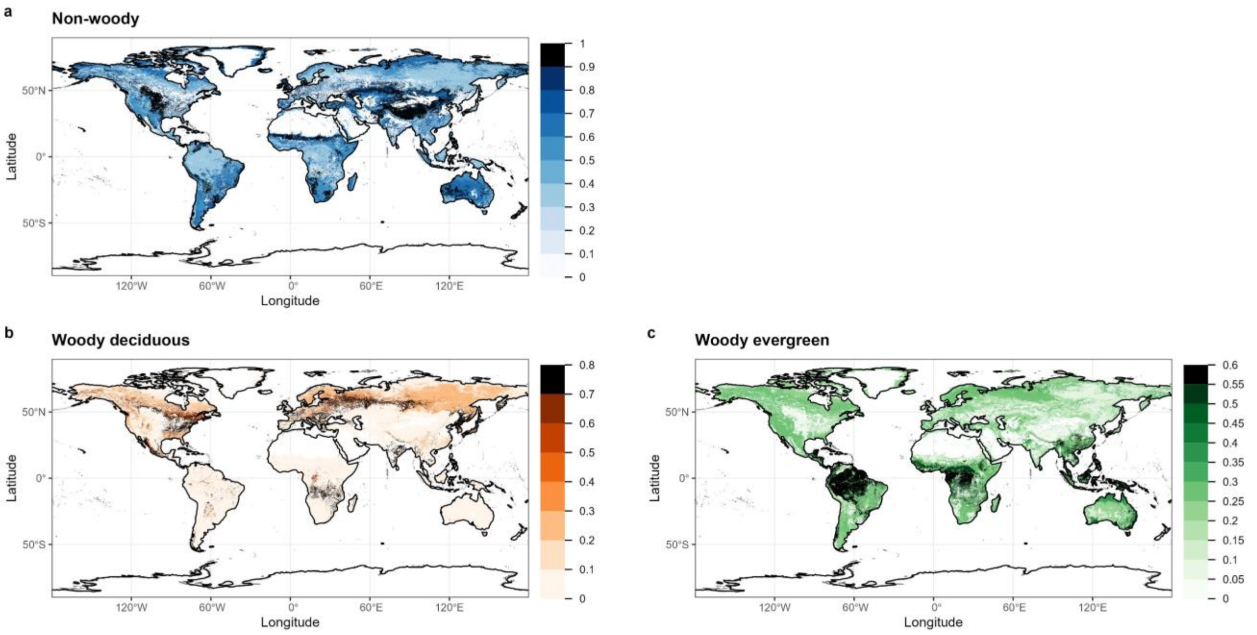

Fig. S5 | Global fractional covers of three plant groups. Global maps of estimated coverage for non-woody plants (**a**), woody deciduous plants (**b**) and woody evergreen plants (**c**). All elements of the figure were created by the authors using R v4.2.2 (https://www.r-project.org/)[82].

Table S6 | Summary of principal component analysis (PCA) for six major plant functional traits in three plant groups. Including trait loadings, eigenvalues, and the proportion of trait variation explained by the first three successive principal components (PC1 – 3). All six traits were natural-log transformed before the analysis. Abbreviations and units of traits are shown in Table 1.

Variables	Non-woody			Woody deciduous			Woody evergreen		
	PC1	PC2	PC3	PC1	PC2	PC3	PC1	PC2	PC3
LA	0.4698	-0.2594	0.3953	-0.4208	0.4291	-0.2152	0.3358	-0.5588	0.0321
LMA	-0.5366	-0.3003	0.2688	-0.2061	-0.6237	-0.1612	0.3440	0.5765	0.0350
N_{area}	-0.4923	-0.2349	0.4974	-0.2510	-0.5759	-0.3588	0.4110	0.5029	0.0511
SSD	-0.4145	-0.1838	-0.6624	-0.3689	-0.2112	0.8494	0.3151	-0.1420	0.8831
H	0.1551	-0.6698	-0.0265	-0.5352	0.1751	-0.2668	0.5046	-0.0684	-0.3877
DM	0.2304	-0.5522	-0.2910	-0.5430	0.1413	0.0796	0.4961	-0.2785	-0.2548
**Eigenvalue**	1.5292	1.1704	0.9324	1.5803	1.4284	0.8655	1.5436	1.3350	0.9421
**Proportion Explained**	0.3898	0.2283	0.1449	0.4162	0.3400	0.1249	0.3971	0.2970	0.1479
**Cumulative Proportion**	0.3898	0.6181	0.7630	0.4162	0.7563	0.8812	0.3971	0.6942	0.8421

Table S7 | Summary of redundancy analysis (RDA) for six major plant functional traits in three plant groups. Including trait loadings, eigenvalues, and the proportion of trait variation explained by three successive redundancy analysis axes (RDA1 – 3) (constrained by climate) and the first three residual principal components (PC1 – 3) (unrelated to climate). All six traits were natural-log transformed before the analysis. Abbreviations and units of traits are shown in Table 1.

Variables	Non-woody						Woody deciduous						Woody evergreen					
	**RDA1**	**RDA2**	**RDA3**	**PC1**	**PC2**	**PC3**	**RDA1**	**RDA2**	**RDA3**	**PC1**	**PC2**	**PC3**	**RDA1**	**RDA2**	**RDA3**	**PC1**	**PC2**	**PC3**
LA	-1.7527	-1.9862	0.6077	6.4243	-4.2646	2.1138	0.6330	3.1101	0.2625	-6.4770	1.8414	1.5128	6.0419	-1.6884	0.3859	-2.7686	4.0152	-0.0701
LMA	1.7818	0.3995	-0.2258	-7.7911	-3.7350	1.2750	4.3021	-1.2954	0.1559	1.5417	-6.1753	1.3505	-1.3114	4.8577	0.3665	-4.4959	-4.8433	0.5587
N_{area}	0.9525	1.4543	0.2692	-7.0363	-3.9918	3.3756	4.5567	-1.2580	0.0525	0.7945	-5.6931	2.7729	-0.0050	5.0672	0.0288	-4.8712	-4.1793	1.6609
SSD	1.9513	1.8881	0.4025	-5.4205	-0.8533	-6.6063	2.7458	-0.1354	-0.1255	-2.9954	-4.0098	-5.8023	5.9326	2.2868	-1.0091	-0.3092	-1.7593	-5.5515
H	3.9885	-1.9976	-0.1889	2.5715	-5.5155	-0.6782	1.9768	1.5890	0.9888	-6.7423	-1.1033	2.0997	1.9235	0.5278	1.6352	-7.2074	1.0865	-1.6330
DM	2.5686	-0.5040	0.4590	3.9751	-5.0898	-3.5118	2.8736	2.2854	-0.9349	-6.1808	-1.0550	-0.3705	3.4255	0.5883	0.2891	-6.1413	3.8639	0.5012
**Eigenvalue**	0.3631	0.1522	0.0098	2.2054	1.1405	0.8020	0.8746	0.3049	0.0289	2.0267	1.3602	0.7367	1.1243	0.7336	0.0514	1.7887	0.9648	0.4658
**Proportion Explained**	0.0605	0.0254	0.0016	0.3676	0.1901	0.1337	0.1458	0.0508	0.0048	0.3378	0.2267	0.1228	0.1874	0.1223	0.0086	0.2981	0.1608	0.0776
**Cumulative Proportion**	0.0605	0.0859	0.0875	0.4551	0.6452	0.7788	0.1458	0.1966	0.2014	0.5392	0.7659	0.8887	0.1874	0.3096	0.3182	0.6163	0.7771	0.8548

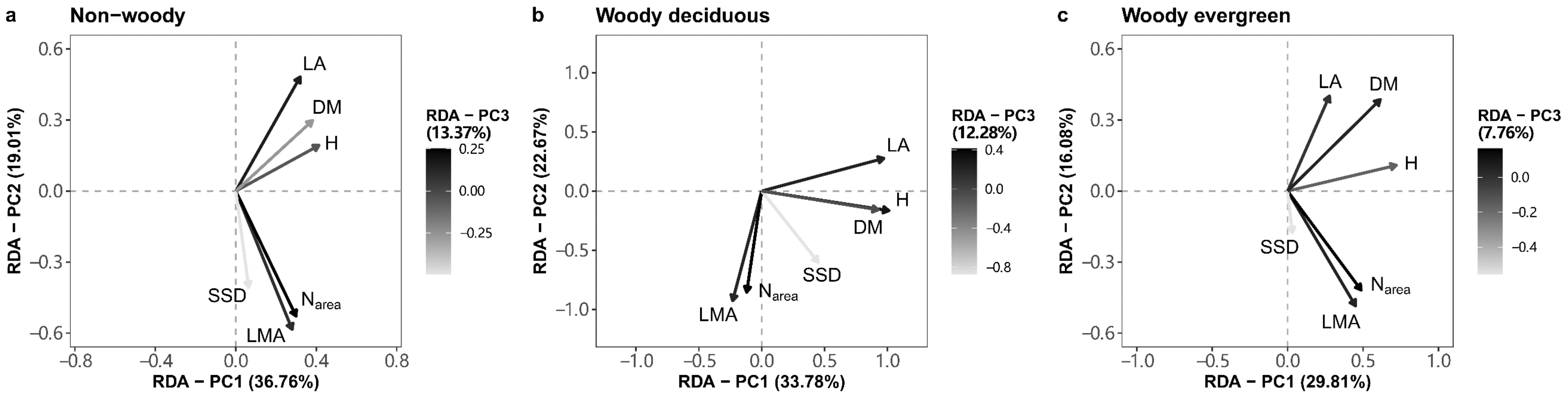

Fig. S6 | Residual (climate-independent) dimensions of trait variation from redundancy analysis for six major plant functional traits of non-woody (a), woody deciduous (b) and woody evergreen (c) plants. The orientation of axes has been rotated according to the Figure 2 in ref. 4. Colour bars indicate the value and direction of the axis 3 for RDA–PC; gray scales indicate the loadings on the third axis. Arrows are upscaled or downscaled to fit the size of the graph. All six traits are natural-log transformed. Natural-log transformed traits were rescaled to a mean of 0 and a standard deviation of 1 before analyses. Abbreviations and units of traits are shown in Table 1. In MI, log-transformed moisture index; MTCO, mean temperature of the coldest month; MGST, mean growing-season temperature (see Methods for definition). All elements of the figure were created by the authors using R v4.2.2 (https://www.r-project.org/)[82].

Table S8 | Summary of generalised additive models (GAMs) based on three bioclimatic variables for six major plant functional traits in three plant groups. Including number of vegetation plots containing community-weighted means (CWMs) of three plant groups for each trait (Sample size), and explained deviance, adjusted R^2 and P values of each GAM model. All six traits were natural-log transformed before the analysis. Abbreviations and units of traits are shown in Table 1.

Trait	Non-woody				Woody deciduous				Woody evergreen			
	**Sample size**	**Deviance explained**	**Adjusted R^2**	***P* value**	**Sample size**	**Deviance explained**	**Adjusted R^2**	***P* value**	**Sample size**	**Deviance explained**	**Adjusted R^2**	***P* value**
LA	60,730	24.10%	0.2406	$< 2 \times 10^{-16}$ ***	39,541	53.12%	0.5309	$< 2 \times 10^{-16}$ ***	46,509	60.64%	0.6062	$< 2 \times 10^{-16}$ ***
LMA	60,872	22.60%	0.2257	$< 2 \times 10^{-16}$ ***	39,197	28.26%	0.2821	$< 2 \times 10^{-16}$ ***	46,432	31.51%	0.3147	$< 2 \times 10^{-16}$ ***
N_{area}	57,520	14.22%	0.1418	$< 2 \times 10^{-16}$ ***	36,509	30.63%	0.3058	$< 2 \times 10^{-16}$ ***	43,537	41.17%	0.4114	$< 2 \times 10^{-16}$ ***
SSD	52,256	23.10%	0.2307	$< 2 \times 10^{-16}$ ***	33,384	17.57%	0.1751	$< 2 \times 10^{-16}$ ***	44,035	57.54%	0.5752	$< 2 \times 10^{-16}$ ***
H	64,114	28.83%	0.2880	$< 2 \times 10^{-16}$ ***	43,528	46.26%	0.4622	$< 2 \times 10^{-16}$ ***	50,953	34.50%	0.3447	$< 2 \times 10^{-16}$ ***
DM	64,211	8.12%	0.0808	$< 2 \times 10^{-16}$ ***	43,146	45.04%	0.4501	$< 2 \times 10^{-16}$ ***	50,182	31.40%	0.3136	$< 2 \times 10^{-16}$ ***

Table S9 | Relative importance of each bioclimatic variable in predicting six major plant functional traits of three plant groups. All six traits were natural-log transformed before the analysis. Abbreviations and units of traits are shown in Table 1. ln MI, log-transformed moisture index; MTCO, mean temperature of the coldest month; MGST, mean growing-season temperature (see Methods for definition).

Trait	Non-woody			Woody deciduous			Woody evergreen		
	ln MI	MTCO	MGST	ln MI	MTCO	MGST	ln MI	MTCO	MGST
LA	64.84%	8.97%	26.19%	62.28%	14.81%	22.91%	3.89%	44.65%	51.46%
LMA	34.65%	19.43%	45.92%	72.60%	3.36%	24.04%	64.13%	24.45%	11.42%
N_{area}	57.92%	13.19%	28.89%	76.19%	3.91%	19.90%	64.10%	15.92%	19.98%
SSD	54.39%	4.82%	40.79%	42.94%	21.70%	35.36%	16.87%	40.33%	42.80%
H	11.79%	25.25%	62.96%	15.34%	21.41%	63.25%	19.17%	19.26%	61.56%
DM	49.47%	15.41%	35.12%	14.05%	44.61%	41.33%	7.68%	29.58%	62.74%

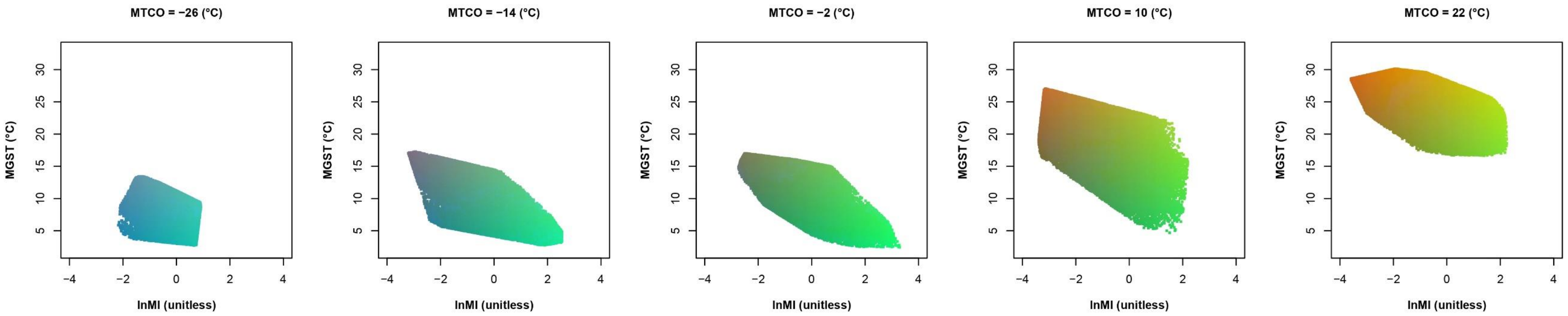

Fig. S7 | Global climate classes in the global climate space. The values of three bioclimatic variables over the globe were rescaled into 0 – 255, respectively, and were then combined as RGB colour codes with red representing mean growing-season temperature (MGST, ℃), green representing natural-log transformed moisture index (ln MI, unitless) and blue representing mean temperature of the coldest month (MTCO, ℃). All elements of the figure were created by the authors using R v4.2.2 (https://www.r-project.org/)[82].

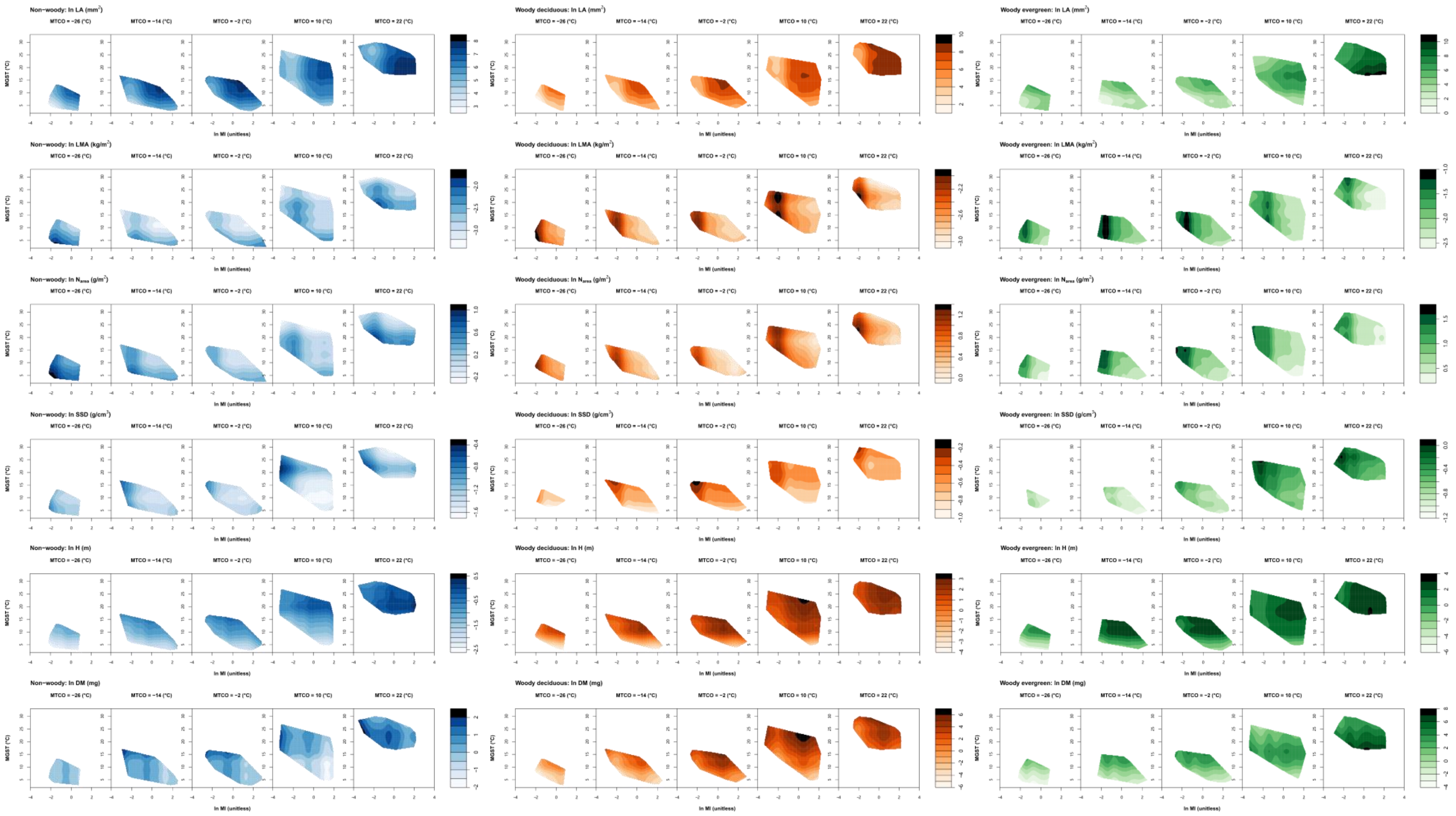

Fig. S8 | Climate space diagrams for six major plant functional traits of non-woody, woody deciduous and woody evergreen plants. Showing distributions of natural-log transformed trait values of three plant groups in the global climate space defined by three bioclimatic variables. Fitted trait values are presented as contours, with darker colours in the right colour bar representing higher trait values. Abbreviations and units of traits are shown

in Table 1. ln MI, log-transformed moisture index; MTCO, mean temperature of the coldest month; MGST, mean growing-season temperature (see Methods for definition). All elements of the figure were created by the authors using R v4.2.2 (https://www.r-project.org/)[82].

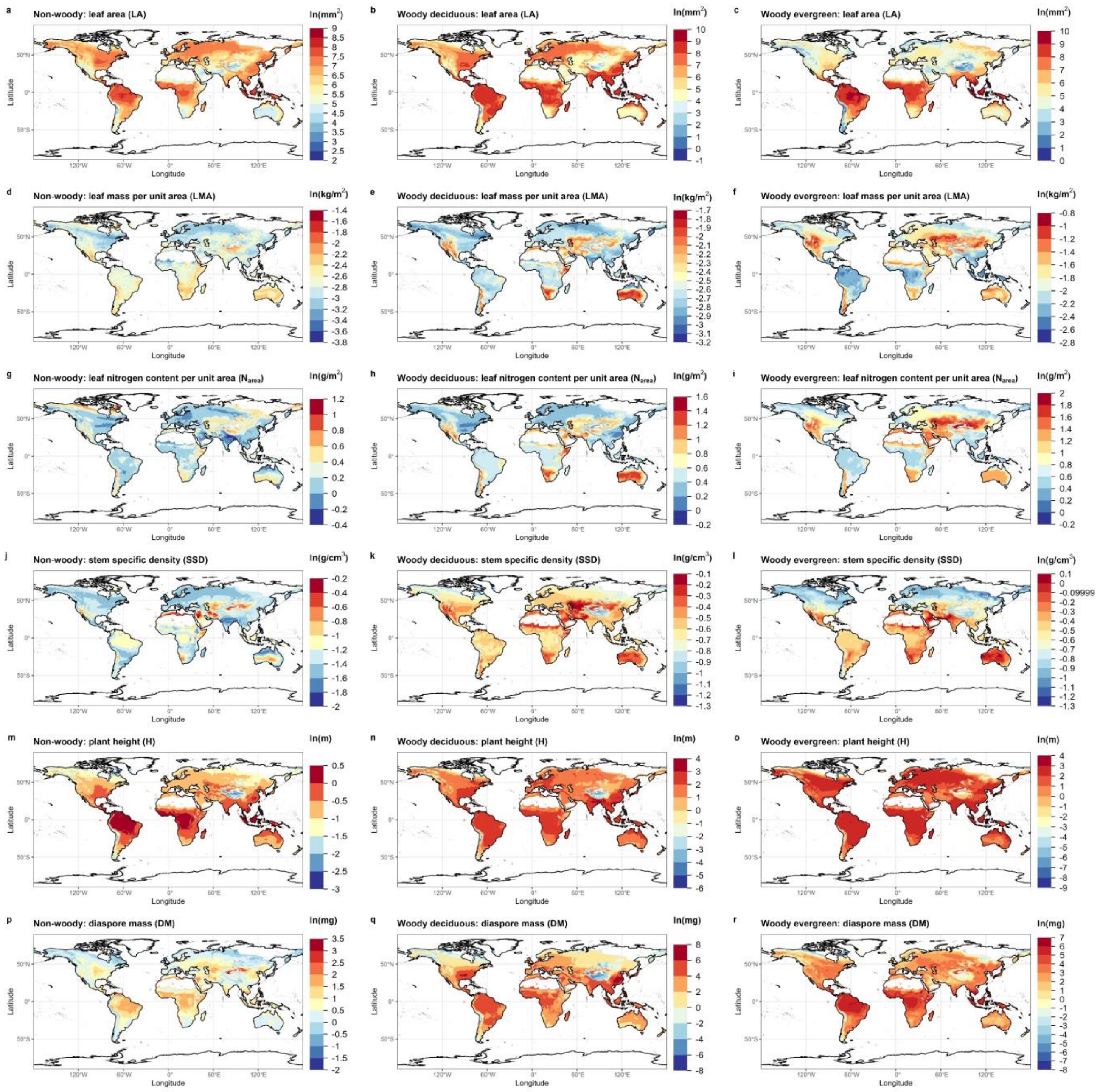

Fig. S9 | Separate global trait maps for six major plant functional traits of three plant groups. Global patterns of natural-log transformed trait values predicted by generalised additive models (GAMs) based on three bioclimatic variables. The maps in the left column are global patterns of the six traits of non-woody plants. The middle-column maps are global patterns of the six traits of woody deciduous plants. The maps in the right column are global patterns of the six traits of woody evergreen plants. All maps are at a 0.1° spatial resolution. For maps in GeoTiff format, refer to the Data availability statement. Abbreviations of traits are shown in Table 1. All elements of the figure were created by the authors using R v4.2.2 (https://www.r-project.org/)[82].

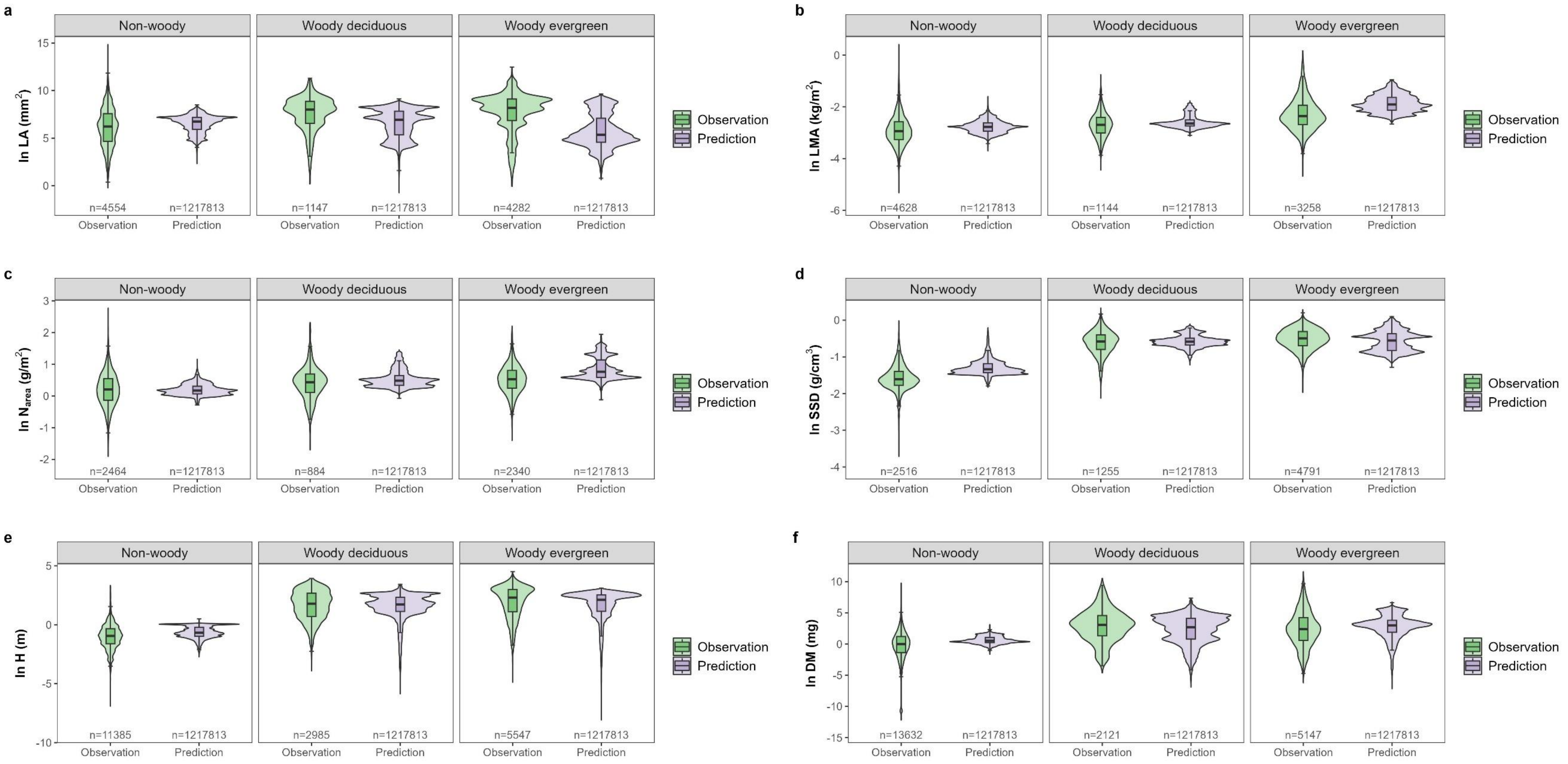

Fig. S10 | Violin plots showing comparisons between observed and predicted values of six major plant functional traits in three plant groups. All six traits are natural-log transformed. Green violin plots show community-weighted means (CWMs) of three plant groups for six traits used to fit

generalised additive models (GAMs). Purple violin plots show pixel values of separate global trait maps for each of the three plant groups. Abbreviations of traits are shown in Table 1. All elements of the figure were created by the authors using R v4.2.2 (https://www.r-project.org/)[82].

Table S10 | Summary of redundancy analysis (RDA) for all 16 plant functional traits. Including trait loadings, eigenvalues, and the proportion of trait variation explained by six successive redundancy analysis axes (RDA1 – 5) (constrained by climate and fractional covers of two of three plant groups). All traits were natural-log transformed before the analysis. Abbreviations and units of traits are shown in Table 1.

Trait	RDA1	RDA2	RDA3	RDA4	RDA5
LA	2.2875	4.1351	0.3359	0.5152	0.3765
LMA	3.7922	-3.2668	-0.5596	-1.6822	0.2021
N_{area}	3.6466	-3.3197	-0.6980	-0.2319	0.2207
SSD	6.3465	-0.5341	-1.0836	-0.1120	-0.5942
H	6.6650	0.3411	0.8414	0.2652	-0.1898
DM	5.7106	0.6421	0.2900	0.5213	-0.2975
LFM	3.3591	3.4485	-0.0480	0.0290	0.2440
P_{area}	0.9985	-4.4006	0.0584	0.9611	0.0768
C_{mass}	3.3403	-0.4903	3.5505	-1.1895	-0.2349
LDMC	3.7144	-1.3566	-0.4824	-0.4554	1.3840
WVL	0.4457	2.5943	1.2475	-1.4273	-0.3608
SCD	-2.1652	-3.6858	3.1341	0.6424	-0.1564
SN	-1.1684	-2.4867	0.2448	0.2550	-0.1338
SL	4.5236	0.4195	0.4516	0.8147	0.0015
DUL	4.2096	0.1319	0.7707	1.3317	0.0602
$\delta^{15}N$	1.9252	-1.3106	-2.7136	-0.1797	-0.8025
**Eigenvalue**	3.3945	1.4603	0.5090	0.1590	0.0514
**Proportion Explained**	0.2122	0.0913	0.0318	0.0099	0.0032
**Cumulative Proportion**	0.2122	0.3034	0.3352	0.3452	0.3484

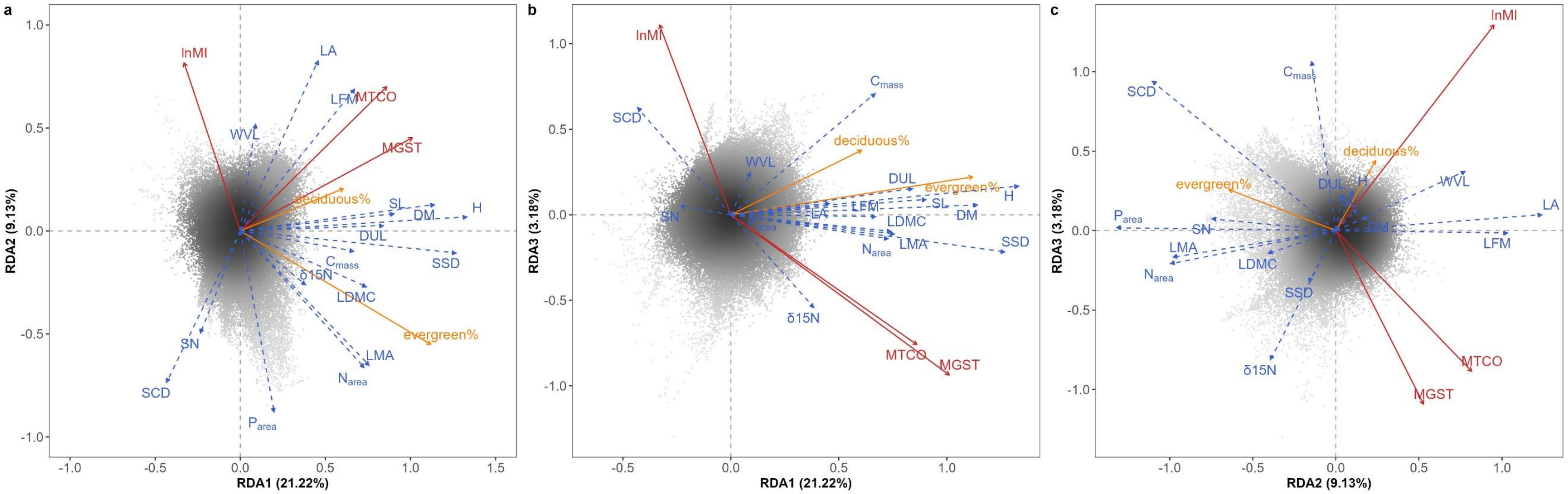

Fig. S11 | Redundancy analysis (RDA) for all 16 plant functional traits. **a**, axis 1 vs axis 2. **b**, axis 1 vs axis 3. **c**, axis 2 vs axis 3. Each point represents a vegetation plot, and the darker colour indicates plot clusters. Correlations among traits and between traits and climates and fractional covers of plant groups are represented by the direction of vectors, where trait vectors are shown in blue arrows, and vectors of bioclimatic variables and fractional covers are shown in red and orange arrows, respectively. Arrows are upscaled or downscaled to fit the size of the graph. As the sum of the global fractional cover for the three plant groups is 1, here only considered the coverage of two of three groups (woody deciduous and woody evergreen). All 16 traits are natural-log transformed. Both bioclimatic variables, fractional covers of plant groups and natural-log transformed traits were rescaled to a mean of 0 and a standard deviation of 1 before analyses. Abbreviations and units of traits are shown in Table 1. ln MI, log-transformed moisture index; MTCO, mean temperature of the coldest month; MGST, mean growing-season temperature (see Methods for definition). deciduous%

and evergreen% are fractional covers of woody deciduous and evergreen plants within a vegetation plot, respectively. All elements of the figure were created by the authors using R v4.2.2 (https://www.r-project.org/)[82].

Table S11 | Summary of the generalised additive models (GAMs) based on three bioclimatic variables and global fractional covers of two of three plant groups for all 16 plant functional traits in 77,074 natural vegetation plots. Including explained deviance, adjusted R^2 and P values of each GAM model. All traits were natural-log transformed before the analysis. Abbreviations and units of traits are shown in Table 1.

Trait	Deviance explained	Adjusted R^2	*P* value
LA	56.50%	0.5640	$< 2 \times 10^{-16}$ ***
LMA	57.53%	0.5743	$< 2 \times 10^{-16}$ ***
N_{area}	49.16%	0.4905	$< 2 \times 10^{-16}$ ***
SSD	67.67%	0.6760	$< 2 \times 10^{-16}$ ***
H	76.75%	0.7669	$< 2 \times 10^{-16}$ ***
DM	63.72%	0.6364	$< 2 \times 10^{-16}$ ***
LFM	54.68%	0.5457	$< 2 \times 10^{-16}$ ***
P_{area}	47.10%	0.4697	$< 2 \times 10^{-16}$ ***
C_{mass}	54.09%	0.5398	$< 2 \times 10^{-16}$ ***
LDMC	39.44%	0.3930	$< 2 \times 10^{-16}$ ***
WVL	32.16%	0.3202	$< 2 \times 10^{-16}$ ***
SCD	61.01%	0.6092	$< 2 \times 10^{-16}$ ***
SN	26.59%	0.2641	$< 2 \times 10^{-16}$ ***
SL	46.23%	0.4611	$< 2 \times 10^{-16}$ ***
DUL	44.93%	0.4481	$< 2 \times 10^{-16}$ ***
$\delta^{15}N$	30.30%	0.3014	$< 2 \times 10^{-16}$ ***

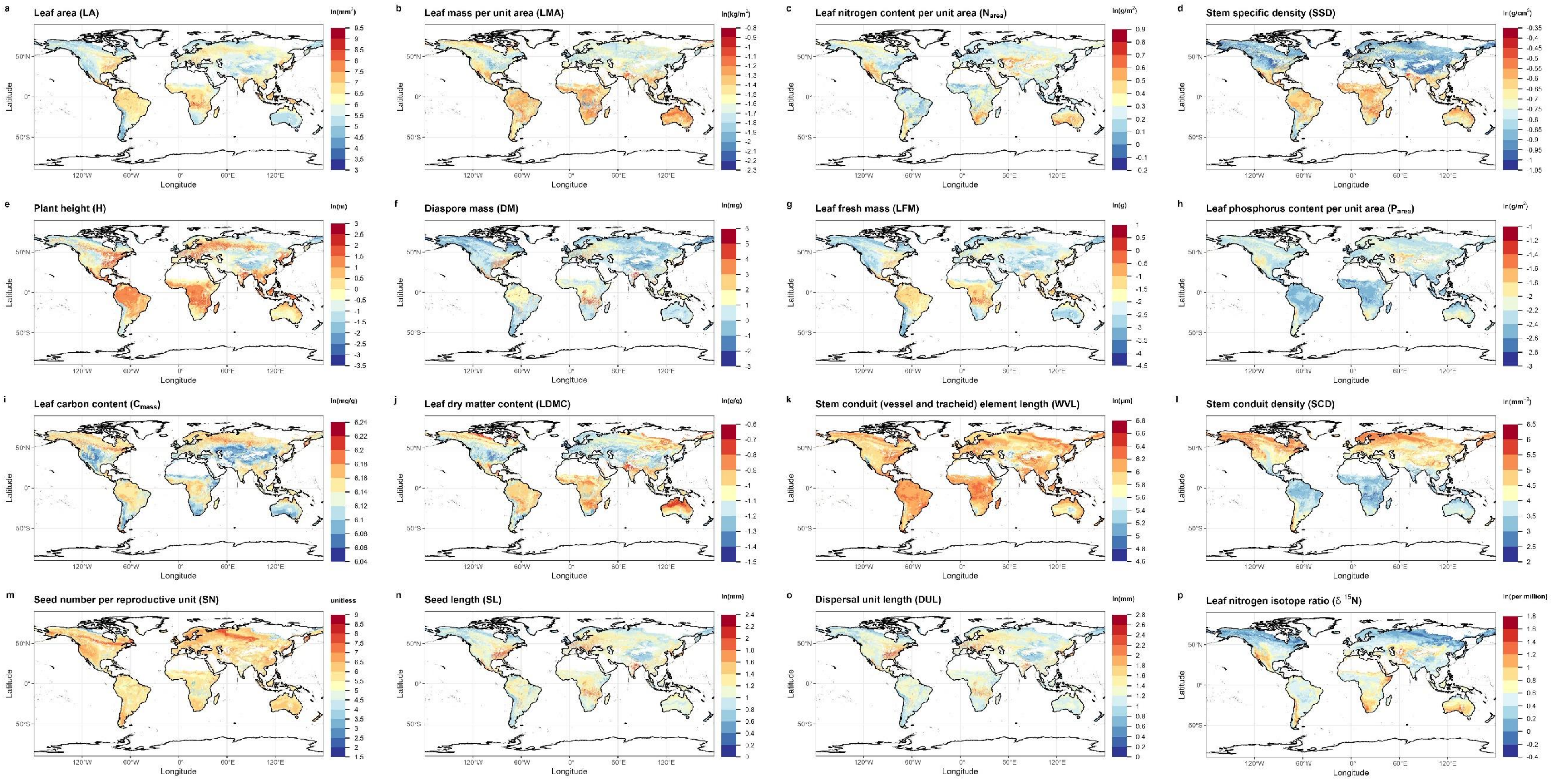

Fig. S12 | Global trait maps for all 16 plant functional traits. Global patterns of natural-log transformed trait values predicted by the generalised additive models (GAMs) based on three bioclimatic variables and global fractional covers of two of three plant groups (see Methods). All maps are at a

0.1° spatial resolution. For maps in GeoTiff format, refer to the Data availability statement. Abbreviations of traits are shown in Table 1. All elements of the figure were created by the authors using R v4.2.2 (https://www.r-project.org/)[82].

Table S12 | Model predictabilities of trait maps in this study and previously published trait maps. R^2_{GAM} represents adjusted R^2 of Generalised additive models (GAMs) in this study. R^2_{Pre} or R_{Pre} indicate adjusted R^2 or correlation coefficient R in previously published studies. Abbreviations and units of traits are shown in Table 1.

<table>
<tr><th rowspan="2">Paper</th><th colspan="2">LA</th><th colspan="2">LMA</th><th colspan="2">N_{area}</th><th colspan="2">SSD</th><th colspan="2">H</th><th colspan="2">DM</th><th colspan="2">LDMC</th></tr>
<tr><th>R^2_{GAM}</th><th>R^2_{Pre}</th><th>R^2_{GAM}</th><th>R^2_{Pre}</th><th>R^2_{GAM}</th><th>R^2_{Pre}</th><th>R^2_{GAM}</th><th>R^2_{Pre}</th><th>R^2_{GAM}</th><th>R^2_{Pre}</th><th>R^2_{GAM}</th><th>R^2_{Pre}</th><th>R^2_{GAM}</th><th>R_{Pre}</th></tr>
<tr><td>Van Bodegom et al. (2014)[5]</td><td rowspan="6">0.5640</td><td>--</td><td rowspan="6">0.5743</td><td>0.3500</td><td rowspan="6">0.4905</td><td>--</td><td rowspan="6">0.6760</td><td>0.5200</td><td rowspan="6">0.7669</td><td>--</td><td rowspan="6">0.6364</td><td>0.4100</td><td rowspan="6">0.3930</td><td>--</td></tr>
<tr><td>Madani et al. (2018)[6]</td><td>--</td><td>--</td><td>--</td><td>--</td><td>0.6620</td><td>0.4550</td><td>--</td></tr>
<tr><td>Moreno-Martínez et al. (2018)[7]</td><td>--</td><td>--</td><td>--</td><td>--</td><td>--</td><td>--</td><td>0.7180</td></tr>
<tr><td>Boonman et al. (2020)[8]</td><td>--</td><td>--</td><td>--</td><td>--</td><td>0.6800</td><td>--</td><td>--</td></tr>
<tr><td>Schiller et al. (2021)[9]</td><td>0.4503</td><td>--</td><td>--</td><td>0.2002</td><td>0.5777</td><td>0.2495</td><td>--</td></tr>
<tr><td>Dong et al. (2023)[10]</td><td>--</td><td>--</td><td>0.2800</td><td>--</td><td>--</td><td>--</td><td>--</td></tr>
</table>

Table S13 | Summary of comparison between model-predicted values and iNaturalist estimated values for all 16 plant functional traits. Including four parameters for assessing agreements between model-predicted map pixel values and the iNaturalist map pixel values. The agreements were estimated by linear regression at a 2° spatial resolution. R^2 is the coefficient of determination; RRMSE is the root-mean-square error, as a proportion of the observed mean trait value (here observations are represented by iNaturalist trait values); bias is the difference between observed and model-predicted mean values, as a proportion of the observed mean trait value (here observed values are iNaturalist trait values); slope is the slope of the linear regression of iNaturalist estimation against model prediction. All traits are natural-log transformed. Abbreviations and units of traits are shown in Table 1.

Trait	R^2	RRMSE	bias	slope
LA	0.36	0.177	-0.0892	1.1
LMA	0.17	-0.249	-0.101	1
N_{area}	0.20	0.81	-0.325	1.45
SSD	0.27	-0.487	-0.245	2.06
H	0.23	6.1	-0.151	0.565
DM	0.13	1.3	-0.622	0.674
LFM	0.36	-0.876	0.419	1.4
P_{area}	0.037	-0.164	0.0171	0.48
C_{mass}	0.045	0.0108	0.00562	0.446
LDMC	0.12	-0.27	-0.209	0.701
WVL	0.062	0.057	0.023	0.464
SCD	0.64	0.234	-0.0566	1.78
SN	0.0091	0.369	-0.25	0.332
SL	0.024	0.729	0.229	0.418
DUL	0.00061	0.77	0.251	0.0656
$\delta^{15}N$	0.24	0.985	-0.268	1.36

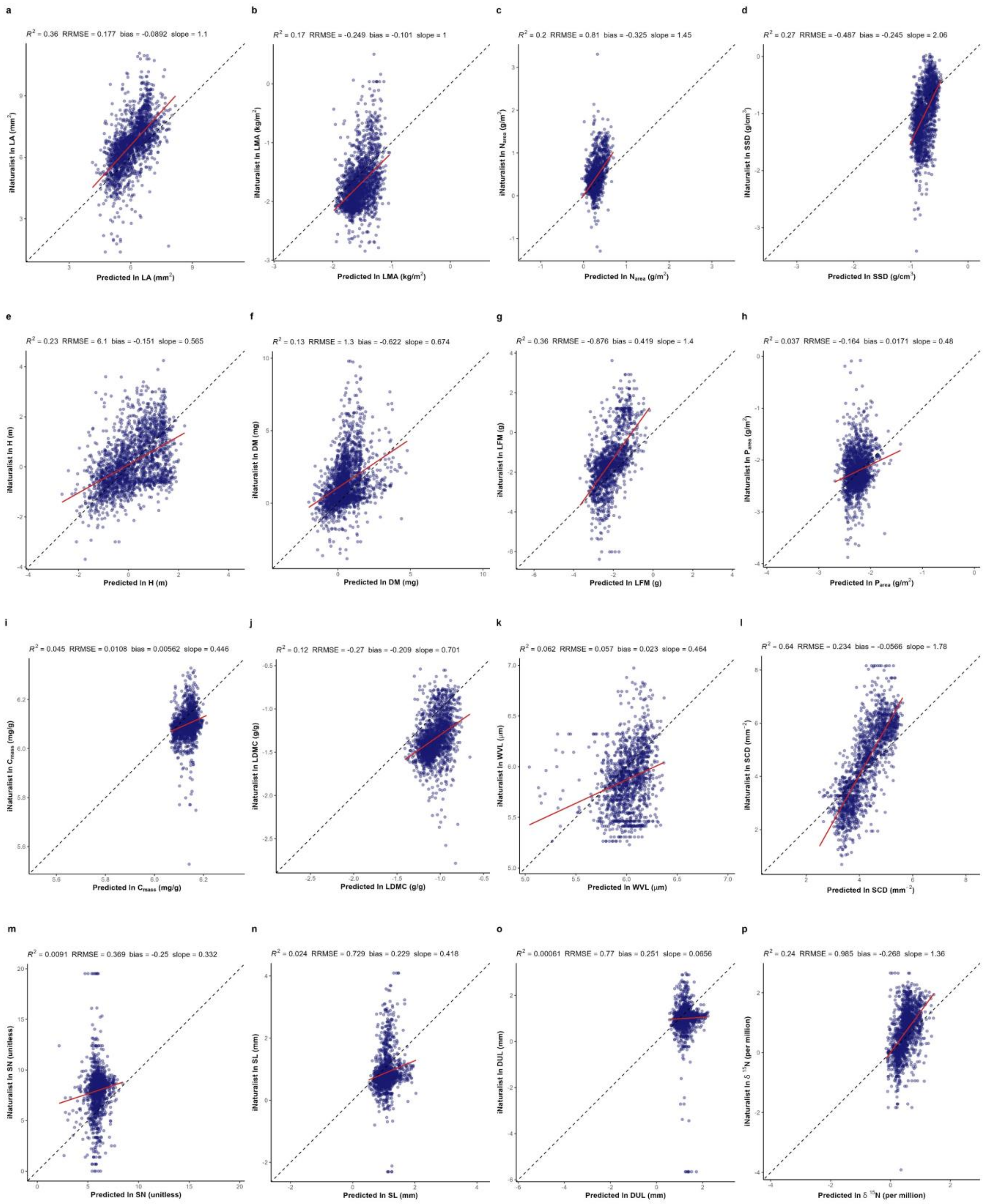

Fig. S13 | Scatter plots of comparison between predicted trait values in this study and estimated trait values from the iNaturalist for all 16 plant functional traits. Red lines visualise the linear regression between model-predicted map pixel values and the iNaturalist map pixel values estimated at a 2° spatial resolution. The dotted line is the 1:1 line. Parameters on each of the above

plots for assessing agreements between global trait maps in this study and the iNaturalist trait maps are listed in Table S13. All traits are natural-log transformed. Abbreviations and units of traits are shown in Table 1. All elements of the figure were created by the authors using R v4.2.2 (https://www.r-project.org/)[82].

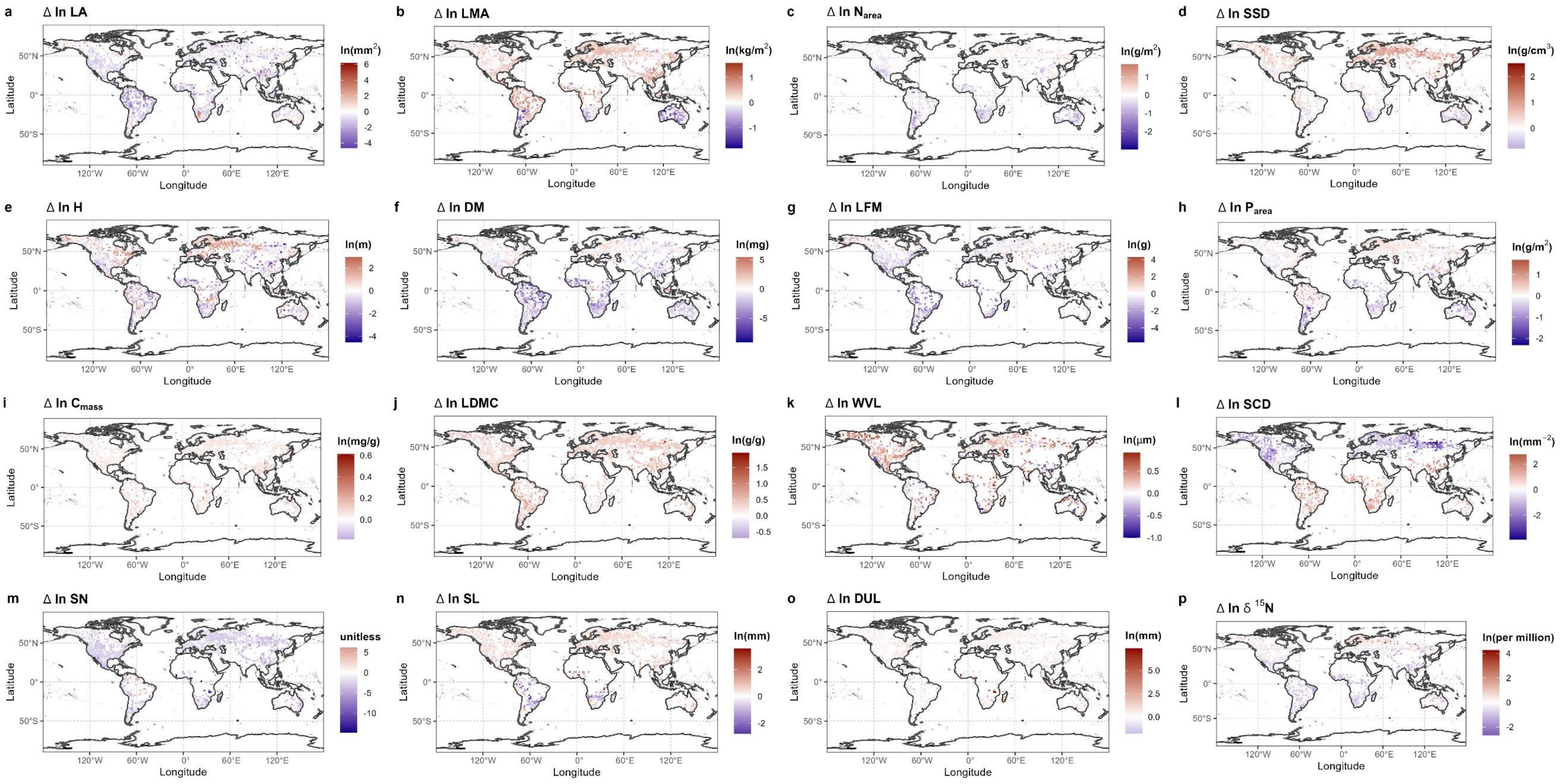

Fig. S14 | Global maps of differentiation between predicted trait values in this study and estimated trait values from the iNaturalist for all 16 plant functional traits at a 2° spatial resolution. Darker colour suggests greater difference between model prediction and iNaturalist estimation. All

traits are natural-log transformed. Abbreviations and units of traits are shown in Table 1. All elements of the figure were created by the authors using R v4.2.2 (https://www.r-project.org/)[82].

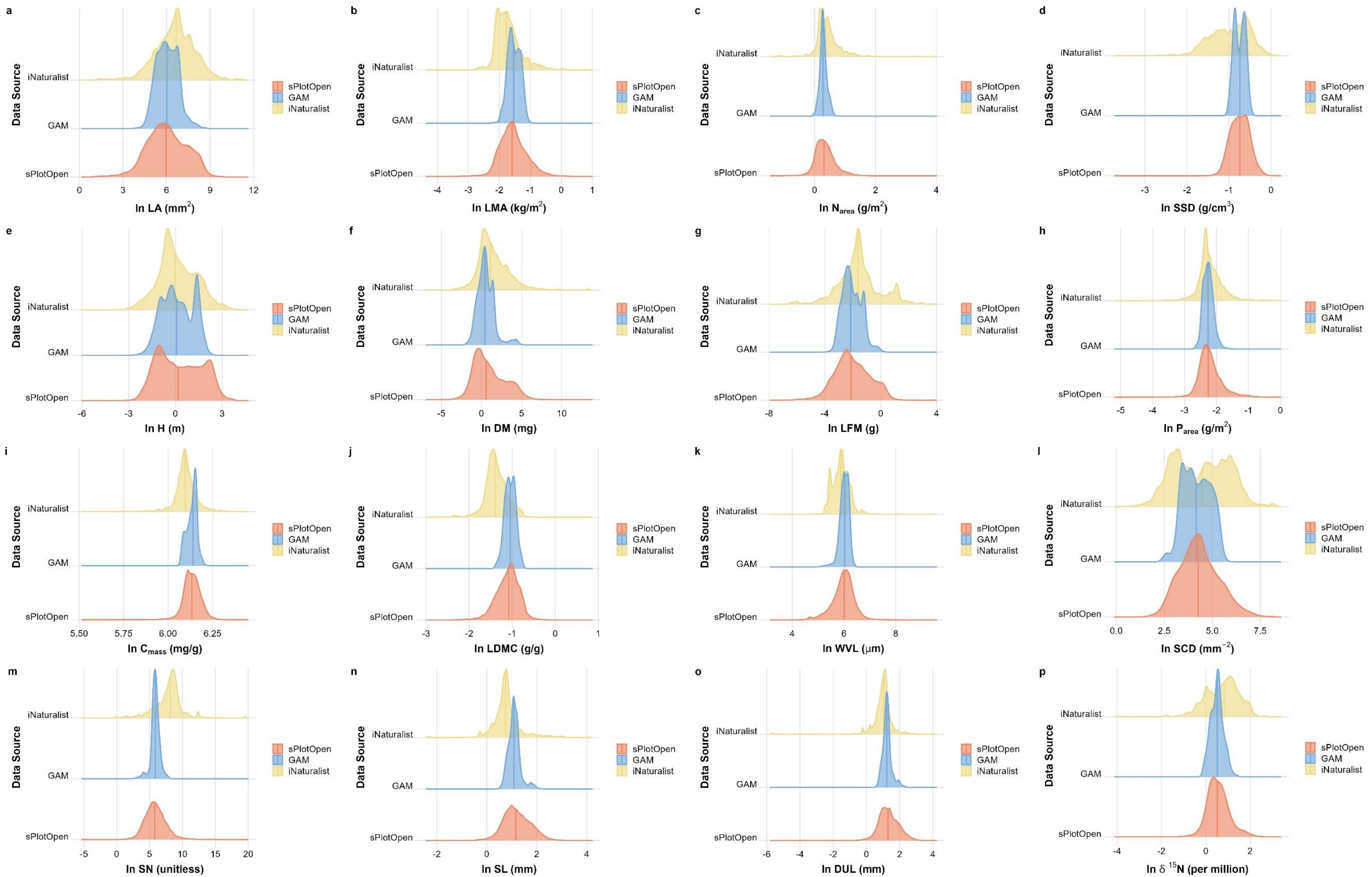
a
b
c
d
e
f
g
h
i
j
k
l
m
n
o
p
Data Source
iNaturalist
GAM
sPlotOpen
ln LA (mm2)
ln LMA (kg/m2)
ln Narea (g/m2)
ln SSD (g/cm3)
ln H (m)
ln DM (mg)
ln LFM (g)
ln Parea (g/m2)
ln Cmass (mg/g)
ln LDMC (g/g)
ln WVL (μm)
ln SCD (mm−2)
ln SN (unitless)
ln SL (mm)
ln DUL (mm)
ln δ 15N (per million)

160

Fig. S15 | Comparisons of trait value distributions among sample trait data based on the sPlotOpen database, model prediction in this study and iNaturalist estimation for all 16 plant functional traits. All traits are natural-log transformed. Abbreviations and units of traits are shown in Table 1. All elements of the figure were created by the authors using R v4.2.2 (https://www.r-project.org/)[82].

Table S14 | Summary of principal component analysis (PCA) for six major plant functional traits in three plant groups based on the global predicted values from the generalised additive modes (GAMs). Including trait loadings, eigenvalues, and the proportion of trait variation explained by the first three successive principal components (PC1 – 3). All six traits are natural-log transformed generated from the GAMs based on bioclimatic variables and global fractional cover of plant groups. Abbreviations and units of traits are shown in Table 1.

Variables	Non-woody			Woody deciduous			Woody evergreen		
	PC1	PC2	PC3	PC1	PC2	PC3	PC1	PC2	PC3
LA	0.5307	0.0385	-0.0086	0.5465	-0.1058	0.3372	0.5496	-0.1457	0.2114
LMA	-0.4379	-0.2658	0.6750	-0.4538	-0.3630	0.2334	-0.2548	0.5839	-0.1700
N_{area}	-0.5260	-0.1662	-0.0762	-0.4386	-0.3486	0.5610	-0.1265	0.6563	0.0151
SSD	-0.2027	-0.5526	-0.5827	-0.1314	-0.5744	-0.6722	0.2975	0.3562	0.7601
H	0.3736	-0.4690	0.4383	0.4116	-0.4124	0.2470	0.4777	0.1890	-0.5461
DM	0.2631	-0.6123	-0.0828	0.3412	-0.4853	-0.0653	0.5480	0.2109	-0.2242
**Eigenvalue**	1.5791	1.3959	0.8900	1.7461	1.5445	0.5658	1.6475	1.4528	0.8879
**Proportion Explained**	0.4156	0.3248	0.1320	0.5082	0.3976	0.0534	0.4524	0.3518	0.1314
**Cumulative Proportion**	0.4156	0.7404	0.8724	0.5082	0.9057	0.9591	0.4524	0.8042	0.9356

Table S15 | Summary of redundancy analysis (RDA) for six major plant functional traits in three plant groups based on the global predicted values from the generalised additive modes (GAMs). Including trait loadings, eigenvalues, and the proportion of trait variation explained by three successive redundancy analysis axes (RDA1 – 3) (constrained by climate) and the first three residual principal components (PC1 – 3) (unrelated to climate). All six traits are natural-log transformed generated from the GAMs based on bioclimatic variables and global fractional cover of plant groups. Abbreviations and units of traits are shown in Table 1.

Variables	Non-woody						Woody deciduous						Woody evergreen					
	RDA1	RDA2	RDA3	PC1	PC2	PC3	RDA1	RDA2	RDA3	PC1	PC2	PC3	RDA1	RDA2	RDA3	PC1	PC2	PC3
LA	-0.9235	15.9216	1.1417	10.5836	-6.8149	4.3864	8.5197	-14.3082	0.6441	12.1757	-0.1670	3.7394	18.8901	-0.2156	2.7081	-2.6101	5.8584	-5.5943
LMA	6.1636	-4.8034	-4.8625	-17.2671	1.4817	7.1244	-14.9008	2.8655	1.1425	-10.2029	10.0346	1.6198	-11.7650	-11.6984	0.9828	-6.3945	-10.3815	-4.1197
N_{area}	-3.9410	-6.4101	1.2422	-17.4243	-7.7709	1.1072	-15.4736	-0.1830	-0.4310	-12.1676	6.0929	4.4843	-8.5521	-16.3188	1.4709	-6.6564	-6.1646	-0.1144
SSD	7.7137	-9.5300	3.0702	-4.4597	-14.2480	-7.6612	-16.0419	-3.9914	-0.9034	3.5148	10.5275	-6.8297	11.0343	-16.2638	-2.1281	2.2477	-2.0315	3.3990
H	17.1876	6.8216	-0.3177	5.0706	-3.7691	5.5367	-0.8075	-14.0303	-2.4601	12.9179	7.0835	4.7074	10.5037	-1.6239	-0.0084	-17.4171	1.4373	4.7707
DM	14.2727	-1.7295	1.2400	6.7665	-12.1366	4.9724	-5.5766	-15.4951	2.0819	11.3135	4.4236	-0.9941	13.4332	-5.7025	-0.2563	-14.0743	4.4426	-2.3977
**Eigenvalue**	1.3606	1.0166	0.0834	1.7872	1.0511	0.4113	1.8271	1.4779	0.0291	1.5707	0.7068	0.2364	2.1773	1.5601	0.0334	1.3285	0.4573	0.1961
**Proportion Explained**	0.2268	0.1694	0.0139	0.2979	0.1752	0.0685	0.3045	0.2463	0.0048	0.2618	0.1178	0.0394	0.3629	0.2600	0.0056	0.2214	0.0762	0.0327
**Cumulative Proportion**	0.2268	0.3962	0.4101	0.7080	0.8831	0.9517	0.3045	0.5508	0.5557	0.8175	0.9353	0.9747	0.3629	0.6229	0.6285	0.8499	0.9261	0.9588

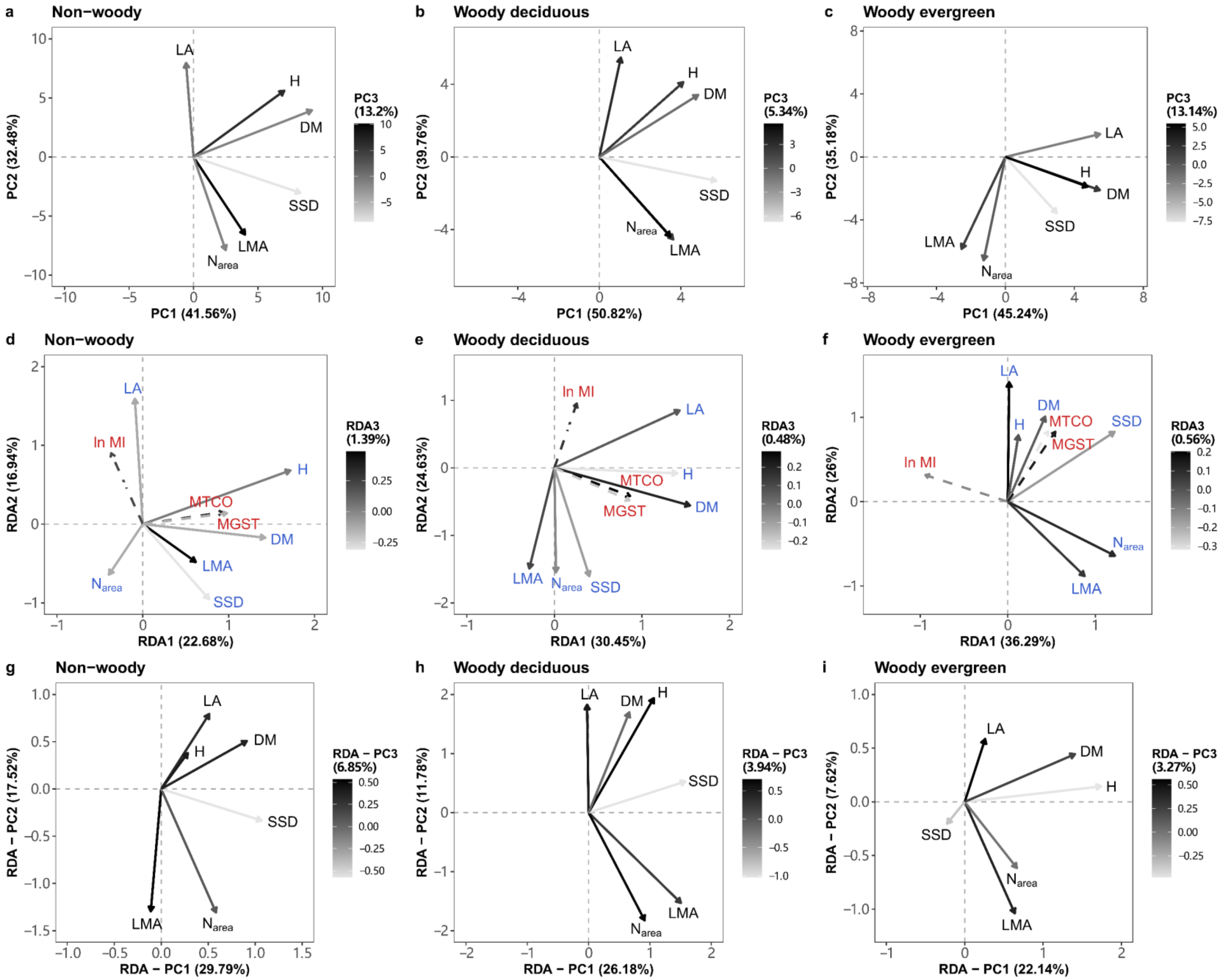
a Non−woody
b Woody deciduous
c Woody evergreen
d Non−woody
e Woody deciduous
f Woody evergreen
g Non−woody
h Woody deciduous
i Woody evergreen
PC1 (41.56%)
PC2 (32.48%)
PC3 (13.2%)
PC1 (50.82%)
PC2 (39.76%)
PC3 (5.34%)
PC1 (45.24%)
PC2 (35.18%)
PC3 (13.14%)
RDA1 (22.68%)
RDA2 (16.94%)
RDA3 (1.39%)
RDA1 (30.45%)
RDA2 (24.63%)
RDA3 (0.48%)
RDA1 (36.29%)
RDA2 (26%)
RDA3 (0.56%)
RDA − PC1 (29.79%)
RDA − PC2 (17.52%)
RDA − PC3 (6.85%)
RDA − PC1 (26.18%)
RDA − PC2 (11.78%)
RDA − PC3 (3.94%)
RDA − PC1 (22.14%)
RDA − PC2 (7.62%)
RDA − PC3 (3.27%)
LA
H
DM
SSD
LMA
Narea
ln MI
MTCO
MGST

Fig. S16 | Principal component analysis (PCA) (a – c), redundancy analysis (RDA) (d – f) and residual (climate-independent) dimensions of trait variation from redundancy analysis (g – i) for six major plant functional traits of non-woody (a, d and g), woody deciduous (b, e and h) and woody evergreen (c, f and i) plants based on the global predicted values from the generalised additive modes (GAMs). The orientation of axes has been rotated according to the Figure 2 in ref. 4. Gray scales indicate the loadings on the third axis. Solid arrows (with blue labels in RDA) represent traits and dashed arrows (with red labels in RDA) represent bioclimatic variables. All six traits are natural-log transformed generated from the GAMs based on bioclimatic variables and global fractional cover of plant groups. Both log-transformed traits and bioclimatic variables were rescaled to a mean of 0 and a standard deviation of 1 before analysis. Abbreviations and units of traits are shown in Table 1. In MI, log-transformed moisture index; MTCO, mean temperature of the coldest month; MGST, mean growing-season temperature (see Methods for definition). All elements of the figure were created by the authors using R v4.2.2 (https://www.r-project.org/)[82].

SI 2 Analysis for mass-based leaf nitrogen content (N_{mass}) and leaf phosphorus content (P_{mass})

Table S16 | Sample size of data used in this study for mass-based leaf nitrogen content (N_{mass}) and leaf phosphorus content (P_{mass}). [a] Plant functional traits used in this study. [b] Abbreviation and [c] unit of each of the 16 traits. [d] Number of plots having plot-level mean trait values. [e] Number of species having species-level mean trait values. [f] Number of pixels of iNaturalist trait maps at 2° spatial resolution. [g–h] Number of pixels of global trait maps upscaled from generalised additive models (GAMs) at [g] 0.1° spatial resolution and [h] 2° spatial resolution. [i] Number of pixels having both iNaturalist trait values and GAM trait values when making comparisons at 2° spatial resolution. N_{mass} has both species-level[1] and plot-level trait[2] means. P_{mass} only have plot-level trait means (community-weighted means, CWMs)[2].

Trait[a]	Abbreviation[b]	Unit[c]	N_{plot}[d]	$N_{species}$[e]	$N_{iNaturalist}$[f]	N_{GAM01}[g]	N_{GAM2}[h]	$N_{Comparison}$[i]
Leaf nitrogen content per unit mass	N_{mass}	mg/g	77,074	35,773	3,568	830,086	2,397	1,924
Leaf phosphorus content per unit mass	P_{mass}	mg/g		0	3,490			1,881

Table S17 | Summary of principal component analysis (PCA) for six major plant functional traits in three plant groups. Including trait loadings, eigenvalues, and the proportion of trait variation explained by the first three successive principal components (PC1 – 3). All six traits were natural-log transformed before the analysis. Abbreviations and units of traits are shown in Table 1.

Variables	Non-woody			Woody deciduous			Woody evergreen		
	PC1	PC2	PC3	PC1	PC2	PC3	PC1	PC2	PC3
LA	0.4998	-0.2762	0.2842	-0.5059	0.3695	-0.1543	0.5494	-0.1859	0.0642
LMA	-0.5141	-0.2084	0.1387	-0.0397	-0.7282	0.2084	0.0064	0.7093	0.0361
N_{mass}	0.4485	0.3251	-0.3971	-0.1885	0.0794	0.9328	0.2025	-0.5937	-0.0853
SSD	-0.4702	-0.1538	-0.5005	-0.2968	-0.5653	-0.2220	0.3370	0.0788	0.8877
H	0.0954	-0.6949	0.2567	-0.5589	0.0555	0.0365	0.4726	0.3127	-0.3668
DM	0.2336	-0.5178	-0.6526	-0.5536	-0.0655	-0.1095	0.5659	0.0769	-0.2545
**Eigenvalue**	1.4888	1.1982	0.9019	1.5796	1.1849	0.9916	1.4941	1.2736	0.9433
**Proportion Explained**	0.3694	0.2393	0.1356	0.4158	0.2340	0.1639	0.3720	0.2703	0.1483
**Cumulative Proportion**	0.3694	0.6087	0.7443	0.4158	0.6498	0.8137	0.3720	0.6424	0.7907

Table S18 | Summary of redundancy analysis (RDA) for six major plant functional traits in three plant groups. Including trait loadings, eigenvalues, and the proportion of trait variation explained by three successive redundancy analysis axes (RDA1 – 3) (constrained by climate) and the first three residual principal components (PC1 – 3) (unrelated to climate). All six traits were natural-log transformed before the analysis. Abbreviations and units of traits are shown in Table 1.

Variables	Non-woody						Woody deciduous						Woody evergreen					
	RDA1	RDA2	RDA3	PC1	PC2	PC3	RDA1	RDA2	RDA3	PC1	PC2	PC3	RDA1	RDA2	RDA3	PC1	PC2	PC3
LA	-1.4986	-2.1922	0.5450	6.7914	-3.7532	2.0324	1.2672	2.9152	0.2329	-6.4083	1.4896	-2.2159	6.1039	-1.4540	0.5490	-3.5106	2.1884	-2.7798
LMA	1.7223	0.6232	-0.1858	-7.0882	-2.7177	0.7665	3.9240	-2.1743	0.2668	1.3469	-3.9399	3.5221	-1.5264	4.7889	0.2231	-3.6463	-4.9494	1.4602
N_{mass}	-1.4116	0.8649	0.1843	6.4414	4.2198	-2.2445	1.9550	-0.6374	-0.2276	-2.1050	4.9285	5.7803	2.2747	-0.9434	-0.8206	1.2113	7.6262	3.0084
SSD	1.6655	2.1140	0.4748	-6.0969	-2.0206	-4.0442	2.6515	-0.6947	-0.0648	-2.8142	-5.8111	2.0031	5.8841	2.5167	-0.9290	-0.1593	-1.6434	3.5972
H	4.2170	-1.4731	-0.1569	2.0779	-6.3099	2.8154	2.2474	1.1576	1.0082	-6.8594	-0.0239	0.3785	1.8677	0.6256	1.6664	-7.4256	0.2394	2.4245
DM	2.5977	-0.1717	0.4880	4.1366	-4.8746	-5.9032	3.3074	1.6100	-0.9043	-6.1223	-1.4226	-0.2379	3.3681	0.6989	0.3593	-6.6437	2.7223	-1.5801
**Eigenvalue**	0.3739	0.1366	0.0092	2.1283	1.1585	0.7463	0.6453	0.2656	0.0297	2.0571	1.1455	0.8086	1.1862	0.4174	0.0604	1.5913	1.229	0.5064
**Proportion Explained**	0.0623	0.0228	0.0015	0.3547	0.1931	0.1244	0.1076	0.0443	0.0049	0.3428	0.1909	0.1348	0.1977	0.0696	0.0101	0.2652	0.2048	0.0844
**Cumulative Proportion**	0.0623	0.0851	0.0866	0.4413	0.6344	0.7588	0.1076	0.1518	0.1568	0.4996	0.6905	0.8253	0.1977	0.2673	0.2773	0.5425	0.7474	0.8318

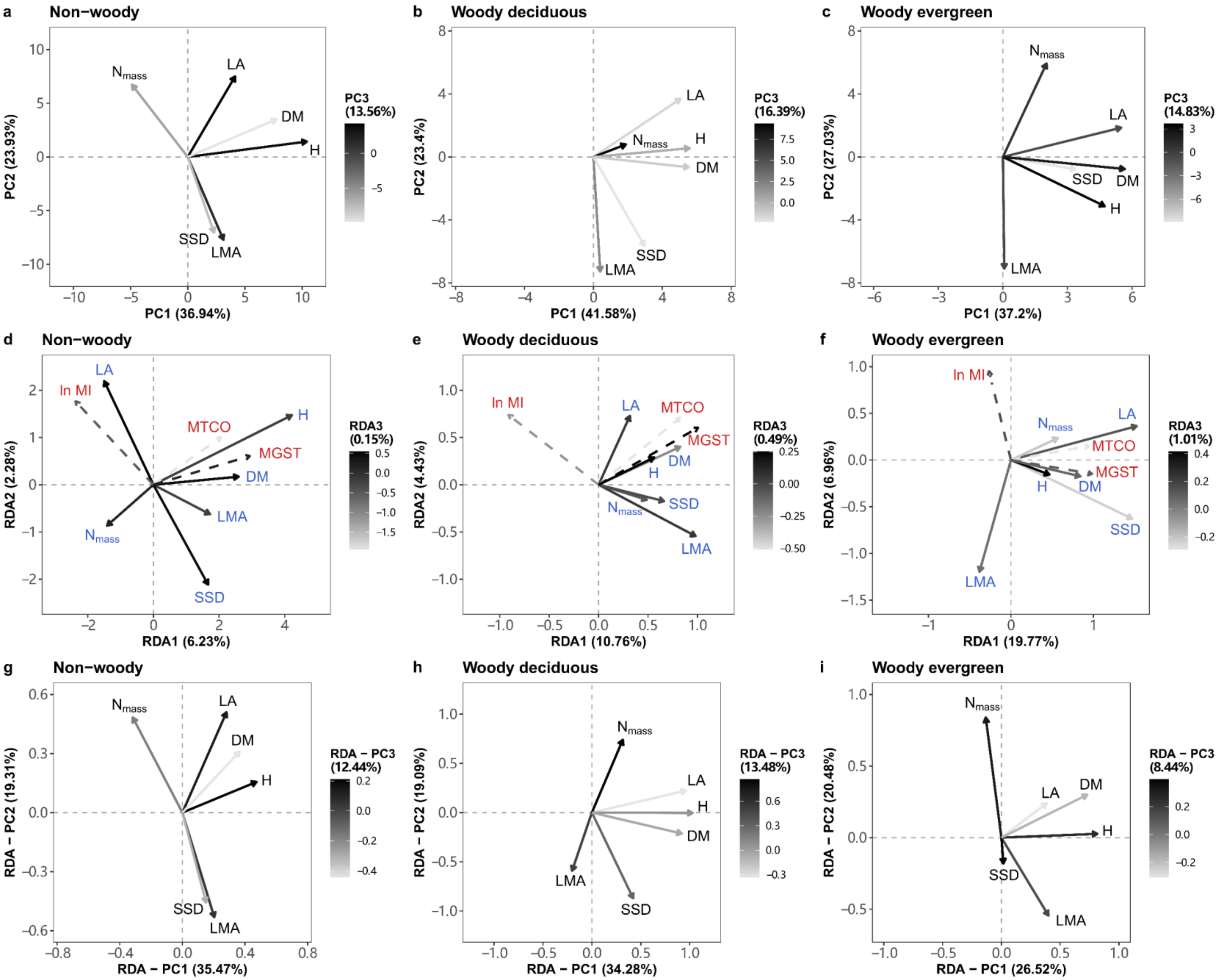
a
Non−woody
N_{mass}
LA
DM
H
SSD
LMA
PC1 (36.94%)
PC2 (23.93%)
PC3 (13.56%)
b
Woody deciduous
LA
N_{mass}
H
DM
SSD
LMA
PC1 (41.58%)
PC2 (23.4%)
PC3 (16.39%)
c
Woody evergreen
N_{mass}
LA
SSD
DM
H
LMA
PC1 (37.2%)
PC2 (27.03%)
PC3 (14.83%)
d
Non−woody
LA
ln MI
MTCO
H
MGST
DM
LMA
N_{mass}
SSD
RDA1 (6.23%)
RDA2 (2.28%)
RDA3 (0.15%)
e
Woody deciduous
ln MI
LA
MTCO
MGST
DM
H
SSD
N_{mass}
LMA
RDA1 (10.76%)
RDA2 (4.43%)
RDA3 (0.49%)
f
Woody evergreen
ln MI
N_{mass}
LA
MTCO
MGST
H
DM
SSD
LMA
RDA1 (19.77%)
RDA2 (6.96%)
RDA3 (1.01%)
g
Non−woody
N_{mass}
LA
DM
H
SSD
LMA
RDA − PC1 (35.47%)
RDA − PC2 (19.31%)
RDA − PC3 (12.44%)
h
Woody deciduous
N_{mass}
LA
H
DM
LMA
SSD
RDA − PC1 (34.28%)
RDA − PC2 (19.09%)
RDA − PC3 (13.48%)
i
Woody evergreen
N_{mass}
LA
DM
H
SSD
LMA
RDA − PC1 (26.52%)
RDA − PC2 (20.48%)
RDA − PC3 (8.44%)

Fig. S17 | Principal component analysis (PCA) (a – c), redundancy analysis (RDA) (d – f) and residual (climate-independent) dimensions of trait variation from redundancy analysis (g – i) for six major plant functional traits of non-woody (a, d and g), woody deciduous (b, e and h) and woody evergreen (c, f and i) plants. The orientation of axes has been rotated according to the Figure 2 in ref. 4. Here we replaced area-based leaf nitrogen content (N_{area}) with mass-based leaf nitrogen content (N_{mass}). Gray scales indicate the loadings on the third axis. Solid arrows (with blue labels in RDA) represent traits and dashed arrows (with red labels in RDA) represent bioclimatic variables. All six traits were natural-log transformed before analysis. Both log-transformed traits and bioclimatic variables were rescaled to a mean of 0 and a standard deviation of 1 before analysis. Abbreviations and units of traits are shown in Table 1. ln MI, log-transformed moisture index; MTCO, mean temperature of the coldest month; MGST, mean growing-season temperature (see Methods for definition). All elements of the figure were created by the authors using R v4.2.2 (https://www.r-project.org/)[82].

Table S19 | Summary of generalised additive models (GAMs) based on three bioclimatic variables for mass-based leaf nitrogen content (N_{mass}) in three plant groups. N_{mass} is natural-log transformed. Including number of vegetation plots containing community-weighted means (CWMs) of three plant groups for each trait (Sample size), and explained deviance, adjusted R^2 and P values of each GAM model.

Plant groups	N_{mass}			
	Sample size	Deviance explained	Adjusted R^2	*P* value
Non-woody	59,964	17.42%	0.1739	$< 2 \times 10^{-16}$ ***
Woody deciduous	38,037	8.98%	0.0892	< 2 × 10-16 ***
Woody evergreen	46,629	16.60%	0.1656	< 2 × 10-16 ***

Table S20 | Relative importance of each bioclimatic variable in predicting mass-based leaf nitrogen content (N_{mass}) of three plant groups. N_{mass} is natural-log transformed. In MI, log-transformed moisture index; MTCO, mean temperature of the coldest month; MGST, mean growing-season temperature (see Methods for definition).

Plant groups	N_{mass}		
	ln MI	MTCO	MGST
Non-woody	11.32%	38.80%	49.88%
Woody deciduous	11.54%	54.70%	33.75%
Woody evergreen	29.47%	38.00%	32.53%

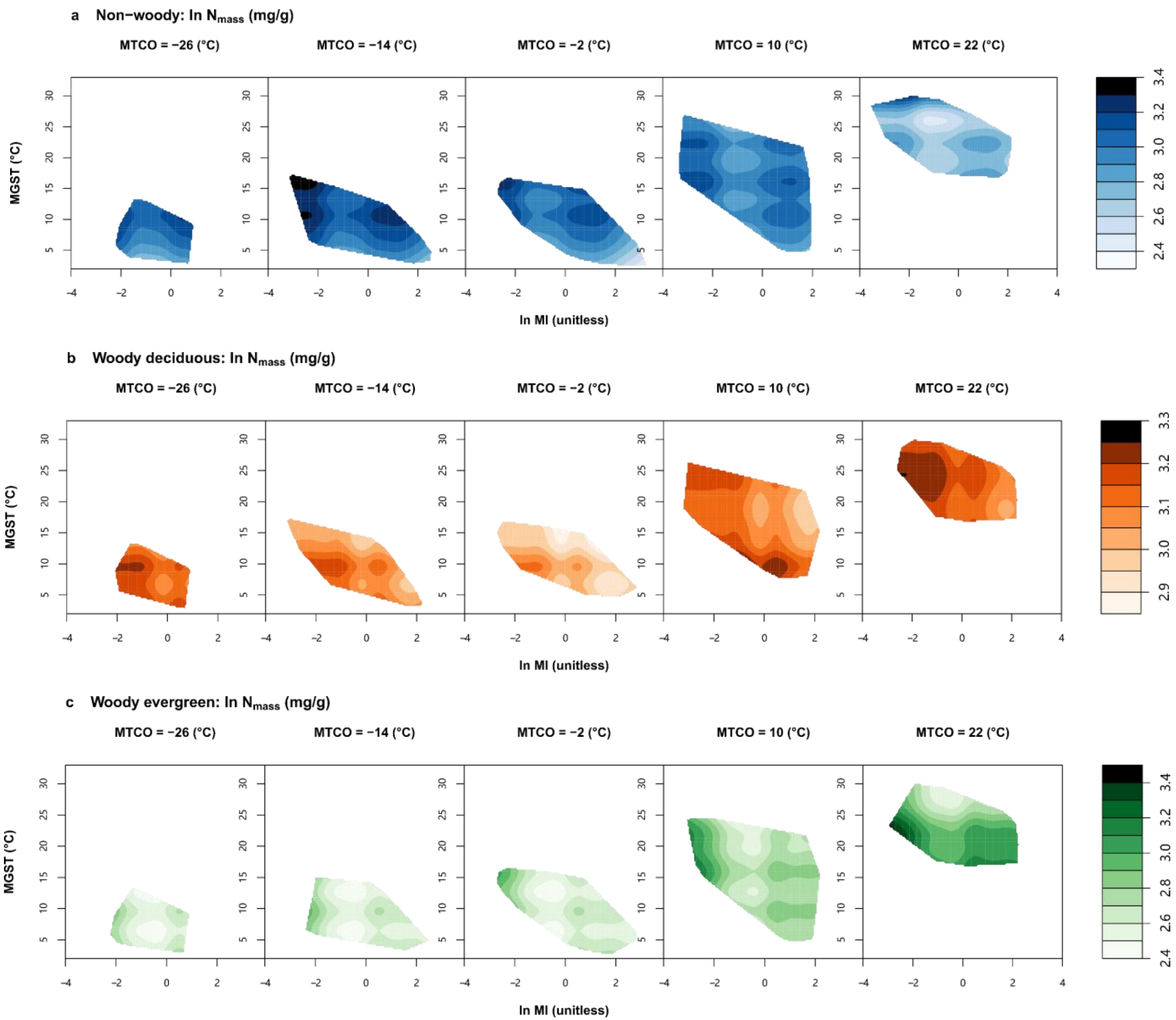

a Non−woody: ln N_{mass} (mg/g)
MTCO = −26 (°C)
MTCO = −14 (°C)
MTCO = −2 (°C)
MTCO = 10 (°C)
MTCO = 22 (°C)
MGST (°C)
ln MI (unitless)
b Woody deciduous: ln N_{mass} (mg/g)
MTCO = −26 (°C)
MTCO = −14 (°C)
MTCO = −2 (°C)
MTCO = 10 (°C)
MTCO = 22 (°C)
MGST (°C)
ln MI (unitless)
c Woody evergreen: ln N_{mass} (mg/g)
MTCO = −26 (°C)
MTCO = −14 (°C)
MTCO = −2 (°C)
MTCO = 10 (°C)
MTCO = 22 (°C)
MGST (°C)
ln MI (unitless)

Fig. S18 | Climate space diagrams of mass-based leaf nitrogen content (N_{mass}) for (a) non-woody, (b) woody deciduous and (c) woody evergreen plants. Showing distributions of natural-log transformed N_{mass} values of three plant groups in the global climate space defined by three bioclimatic variables. The predicted trait values are presented as contours with darker colour representing higher values. In MI, natural-log transformed; MTCO, mean temperature of the coldest month; MGST, mean growing-season temperature (see Methods for definition). All elements of the figure were created by the authors using R v4.2.2 (https://www.r-project.org/)[82].

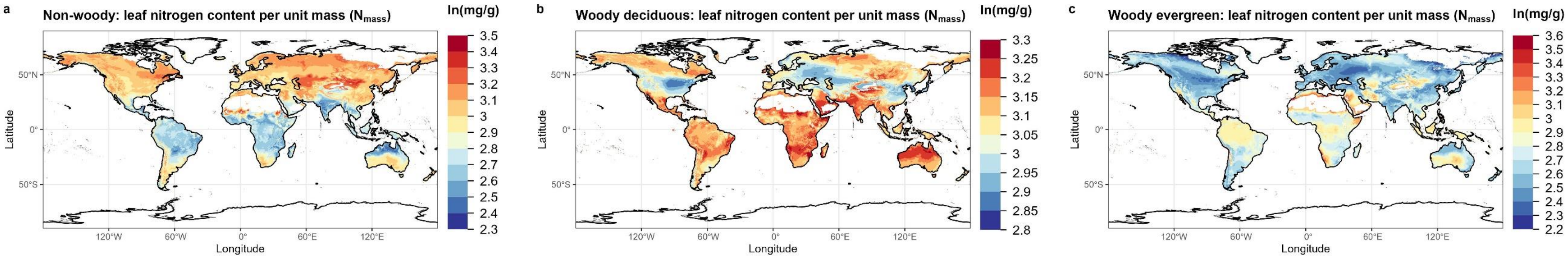

Fig. S19 | Separate global trait maps of mass-based leaf nitrogen content (N_{mass}) for non-woody (a), woody deciduous (b) and woody evergreen (c) plants. Global patterns of natural-log transformed N_{mass} values predicted by generalised additive models (GAMs) based on three bioclimatic variables. All maps are at a 0.1° spatial resolution. For maps in GeoTiff format, refer to the Data availability statement. All elements of the figure were created by the authors using R v4.2.2 (https://www.r-project.org/)[82].

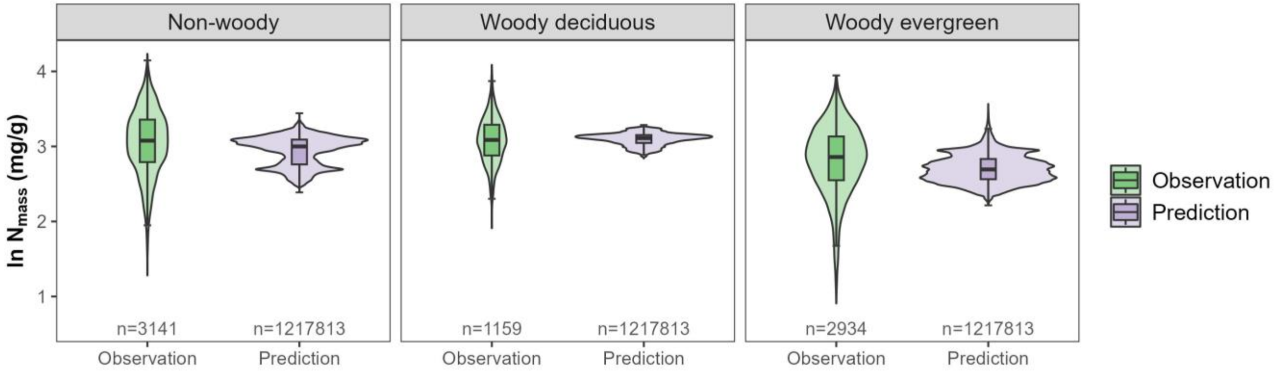

Fig. S20 | Violin plots showing comparisons between observed and predicted values of mass-based leaf nitrogen content (N_{mass}) in three plant groups. N_{mass} is natural-log transformed. Green violin plots show community-weighted means (CWMs) of N_{mass} in three plant groups to fit generalised additive models (GAMs). Purple violin plots show pixel values of separate global trait maps for each of the three plant groups. All elements of the figure were created by the authors using R v4.2.2 (https://www.r-project.org/)[82].

Table S21 | Summary of the generalised additive models (GAMs) based on three bioclimatic variables and global fractional covers of two of three plant groups for mass-based leaf nitrogen content (N_{mass}) and leaf phosphorus content (P_{mass}) in 77,074 natural vegetation plots. N_{mass} and P_{mass} were natural-log transformed before analysis. Including explained deviance, adjusted R^2 and P values of each GAM model.

Trait	Deviance explained	Adjusted R^2	*P* value
N_{mass}	44.78%	0.4464	$< 2 \times 10^{-16}$ ***
P_{mass}	48.60%	0.4847	$< 2 \times 10^{-16}$ ***

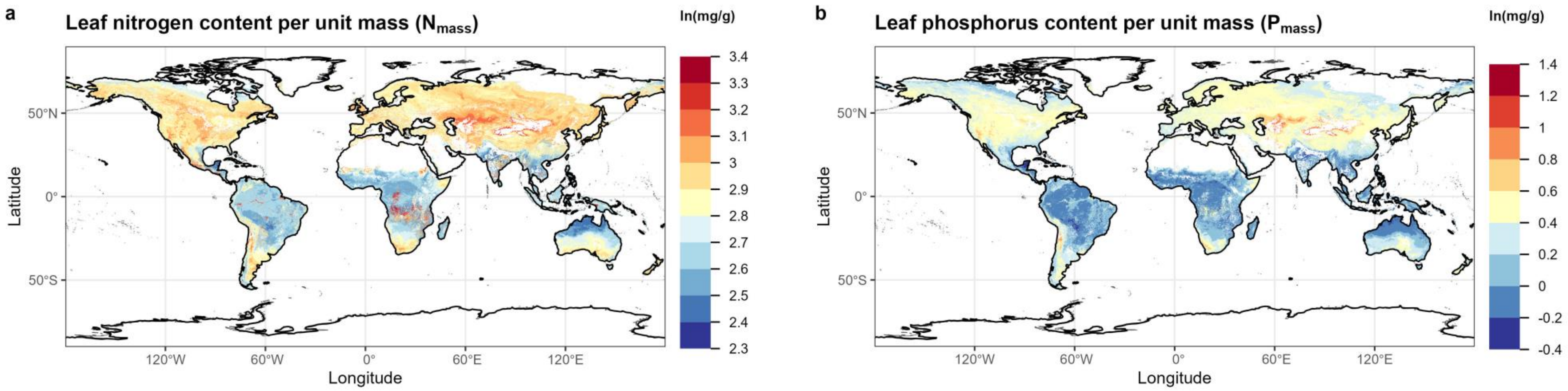

Fig. S21 | Global trait maps of mass-based leaf nitrogen content (N_{mass}, a) and mass-based leaf phosphorus content (P_{mass}, b). Global patterns of natural-log transformed trait values predicted by the generalised additive models (GAMs) based on three bioclimatic variables and global fractional covers of two of three plant groups (see Methods). Both maps are at a 0.1° spatial resolution. For maps in GeoTiff format, refer to the Data availability statement. All elements of the figure were created by the authors using R v4.2.2 (https://www.r-project.org/)[82].

Table S22 | Summary of comparison between model-predicted values and iNaturalist estimated values of mass-based leaf nitrogen content (N_{mass}) and leaf phosphorus content (P_{mass}). Including parameters for assessing agreements between model-predicted map pixel values and the iNaturalist map pixel values. The agreements were estimated by linear regression at a 2° spatial resolution. R^2 is the coefficient of determination; RRMSE is the root-mean-square error, as a proportion of the observed mean trait value (here observations are represented by iNaturalist trait values); bias is the difference between observed and model-predicted mean values, as a proportion of the observed mean trait value (here observed values are iNaturalist trait values); slope is the slope of the linear regression of iNaturalist estimation against model prediction. Both traits are natural-log transformed.

Trait	R^2	RRMSE	bias	slope
N_{mass}	0.024	0.105	-0.0744	0.21
P_{mass}	0.071	0.769	-0.43	0.373

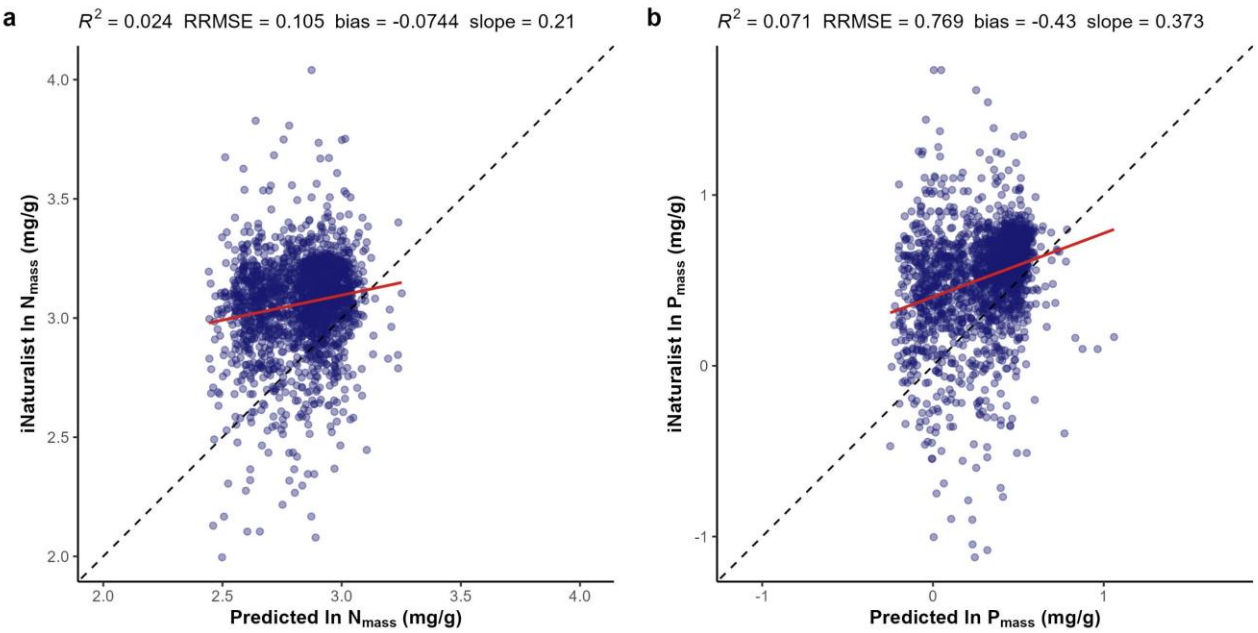

Fig. S22 | Scatter plots of comparison between predicted trait values in this study and estimated trait values from the iNaturalist for mass-based leaf nitrogen content (N_{mass}, a) and mass-based leaf phosphorus content (P_{mass}, b). Red lines represent correlations between model-predicted map pixel values and the iNaturalist map pixel values estimated by linear regression at a 2° spatial resolution. The dotted line is the 1:1 line. Parameters on each of the above plots for assessing agreements between global trait maps in this study and the iNaturalist trait maps are listed in the caption of the Table S13. Both traits are natural-log transformed. All elements of the figure were created by the authors using R v4.2.2 (https://www.r-project.org/)[82].

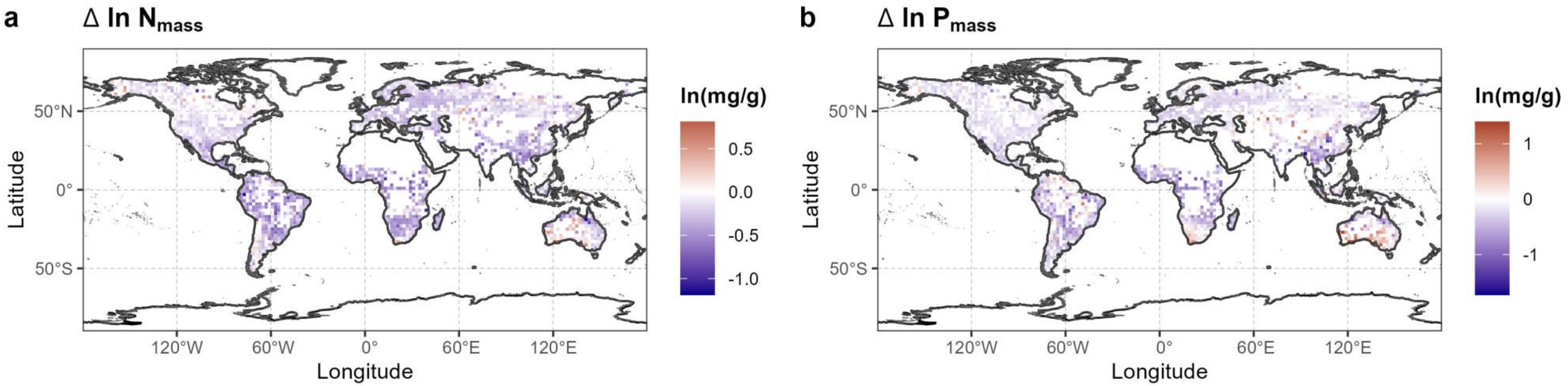

Fig. S23 | Global maps of differentiation between predicted trait values in this study and estimated trait values from the iNaturalist at a 2° spatial resolution. a, mass-based leaf nitrogen content (N_{mass}) and **b**, mass-based leaf phosphorus content (P_{mass}). Darker colour suggests greater difference between model prediction and iNaturalist estimation. Both traits are natural-log transformed. All elements of the figure were created by the authors using R v4.2.2 (https://www.r-project.org/)[82].

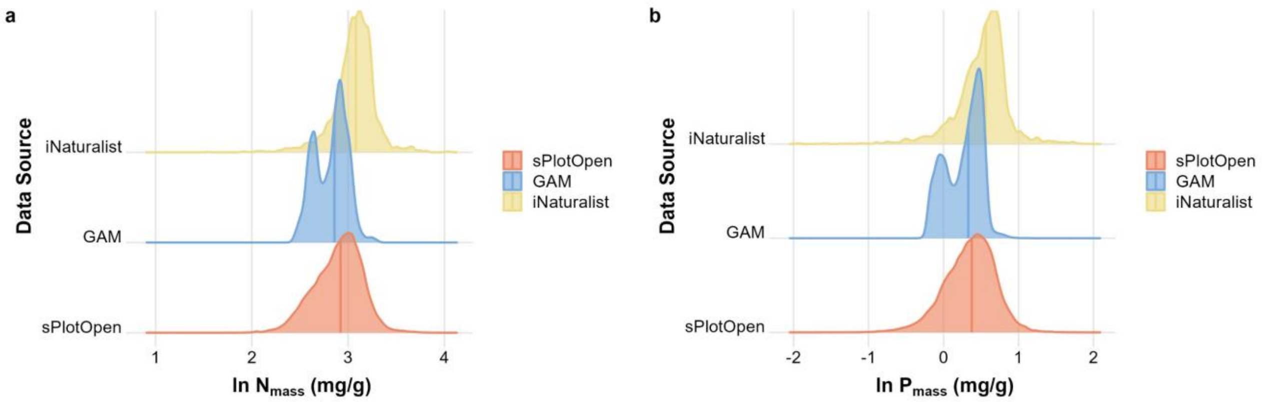

Fig. S24 | Comparisons of trait value distributions among sample trait data based on the sPlotOpen database, model prediction in this study and iNaturalist estimation for mass-based leaf nitrogen content (N_{mass}, a) and mass-based leaf phosphorus content (P_{mass}, b). Both traits are natural-log transformed. All elements of the figure were created by the authors using R v4.2.2 (https://www.r-project.org/)[82].

References

1. Díaz, S. *et al.* The global spectrum of plant form and function: enhanced species-level trait dataset. *Sci. Data* **9**, 755 (2022).
2. Sabatini, F. M. *et al.* sPlotOpen-An environmentally balanced, open-access, global dataset of vegetation plots. *Glob. Ecol. Biogeogr.* **30**, 1740–1764 (2021).
3. Hijmans, R. J. *et al.* Package ‘raster’. *R package* vol. 734 473 (2015).
4. Díaz, S. *et al.* The global spectrum of plant form and function. *Nature* **529**, 167–171 (2016).
5. Van Bodegom, P. M., Douma, J. C. & Verheijen, L. M. A fully traits-based approach to modeling global vegetation distribution. *Proc. Natl. Acad. Sci.* **111**, 13733–13738 (2014).
6. Madani, N. *et al.* Future global productivity will be affected by plant trait response to climate. *Sci. Rep.* **8**, 1–10 (2018).
7. Moreno-Martínez, Á. *et al.* A methodology to derive global maps of leaf traits using remote sensing and climate data. *Remote Sens. Environ.* **218**, 69–88 (2018).
8. Boonman, C. C. F. *et al.* Assessing the reliability of predicted plant trait distributions at the global scale. *Glob. Ecol. Biogeogr.* **29**, 1034–1051 (2020).

9. Schiller, C., Schmidtlein, S., Boonman, C., Moreno-Martínez, A. & Kattenborn, T. Deep learning and citizen science enable automated plant trait predictions from photographs. *Sci. Rep.* **11**, 16395 (2021).

10. Dong, N., Dechant, B., Wang, H., Wright, I. J. & Prentice, I. C. Global leaf-trait mapping based on optimality theory. *Glob. Ecol. Biogeogr.* (2023).
